# Distinguishing sleep from wake with a radar sensor: a contact-free real-time sleep monitor

**DOI:** 10.1093/sleep/zsab060

**Published:** 2021-01-08

**Authors:** Hanne Siri Amdahl Heglum, Håvard Kallestad, Daniel Vethe, Knut Langsrud, Trond Sand, Morten Engstrøm

**Affiliations:** 1 Department of Neuromedicine and Movement Science, Norwegian University of Science and Technology, Trondheim, Norway; 2 Novelda AS, Trondheim, Norway; 3 Department of Neurology and Clinical Neurophysiology, St. Olavs University Hospital, Trondheim, Norway; 4 Department of Mental Health, Norwegian University of Science and Technology, Trondheim, Norway; 5 Division of Mental Health Care, St. Olavs University Hospital, Trondheim, Norway

**Keywords:** sleep, radar, actigraphy, polysomnography, sleep monitoring, ambulatory home monitoring

## Abstract

This work aimed to evaluate whether a radar sensor can distinguish sleep from wakefulness in real time. The sensor detects body movements without direct physical contact with the subject and can be embedded in the roof of a hospital room for completely unobtrusive monitoring. We conducted simultaneous recordings with polysomnography, actigraphy, and radar on two groups: healthy young adults (*n* = 12, four nights per participant) and patients referred to a sleep examination (*n* = 28, one night per participant). We developed models for sleep/wake classification based on principles commonly used by actigraphy, including real-time models, and tested them on both datasets. We estimated a set of commonly reported sleep parameters from these data, including total-sleep-time, sleep-onset-latency, sleep-efficiency, and wake-after-sleep-onset, and evaluated the inter-method reliability of these estimates. Classification results were on-par with, or exceeding, those often seen for actigraphy. For real-time models in healthy young adults, accuracies were above 92%, sensitivities above 95%, specificities above 83%, and all Cohen's kappa values were above 0.81 compared to polysomnography. For patients referred to a sleep examination, accuracies were above 81%, sensitivities about 89%, specificities above 53%, and Cohen's kappa values above 0.44. Sleep variable estimates showed no significant intermethod bias, but the limits of agreement were quite wide for the group of patients referred to a sleep examination. Our results indicate that the radar has the potential to offer the benefits of contact-free real-time monitoring of sleep, both for in-patients and for ambulatory home monitoring.

**Statement of Significance**

This work shows that a contact-free radar sensor with our real-time actigraphy-inspired algorithm can detect body movements and provide valid estimates of sleep, wakefulness, and related parameters. The performance was best for healthy volunteers in a psychiatric hospital environment, with the radar placed either on the nightstand or permanently mounted in the ceiling. The radar generally performed on par with actigraphy for sleep/wake classification. Contact-free recording is an advantage for patients with less tolerance for wearable devices, e.g. in psychiatric hospitals. Future studies should attempt to improve the performance for home use and further validate this tool for sleep/wake classification in a wider population, particularly in a real in-hospital setting. Sleep-stage classification from radar data should also be explored.

## Introduction

The polysomnography (PSG) test [1] is considered the gold standard of sleep studies, but it is not always an ideal option [2, 3]. The multiple on-body sensors can be experienced as invasive and uncomfortable, and the time and expense involved in laborious manual interpretation make it a poor choice for long-term monitoring or population studies. Fortunately, electrophysiological signals are not the only way to get a measure of sleep. Body movement modalities have been used for this purpose since the first actigraphs were validated in the late 1970s–early 1980s [4–6]. These devices typically record activity with an on-body accelerometer and determine activity–rest from those signals, commonly through simple automatic classification models [7, 8]. Compared with PSG, they are cost-effective, noninvasive, reasonably reliable for estimating periods of sleep (although also recognized as having unfortunately low specificity for periods of wakefulness during the night), and particularly well suited for monitoring activity–rest cycles over time. These properties have granted them a position of ubiquity in sleep medicine and research as an alternative when PSG is not feasible or desirable [9–11].

However, sometimes even a wrist actigraph can be too demanding. For patients in a psychiatric hospital setting, with, for example, disorganized behavior, psychosis, or suicidal intents, the use of on-body sensor equipment of any kind can be challenging, and potentially dangerous. And although actigraphy data are collected prospectively, they are analyzed retrospectively, so they can generally not be used to monitor sleep in real time. Thus, they are less useful for hospital night staff who needs to keep track of the sleep/wake state of patients at any given time. So, even though sleep problems are virtually ubiquitous among inpatients with mental disorders [12], and although assessment of sleep can be crucial in diagnosis and decision making, psychiatric units typically remain limited to intermittent staff observations. This solution is far from ideal; the reliability is limited, and it has been argued that the downsides in terms of sleep disruption and privacy infringement for the patients might outweigh the benefits of this type of monitoring by a fairly wide margin [13].

This work seeks to evaluate if a radar sensor can be used to provide an unobtrusive contact-free objective measure of sleep. The sensor in question, an Impulse-Radio Ultra-Wideband (IR-UWB) radar, can detect a range of movements from a person in a room without requiring them to wear equipment on their body; from the big movements of limbs to the very small motions induced by respiration. Under favorable conditions, even the miniscule chest movements caused by a beating heart can be recognized and recorded [14, 15]. Clothes or beddings do not impede these signals [16], and since the registered data cannot be used to directly identify individuals, a degree of privacy is preserved. The radar is also capable of communicating its signals in real time.

The general aim of the present study was to evaluate whether the body movement-derived signals recorded with the radar sensor can be used to distinguish sleep from wake at least as well as the activity signals from wrist actigraphy. In addition, we aimed to evaluate whether contact-free sleep monitoring can be done in real time. Our main objectives were to develop sleep/wake classification models for the radar data, including real-time models, and to compare the classification performance of the radar with PSG (the gold standard) and actigraphy. Models were first applied to a homogenous dataset from young healthy adults recorded in a hospital environment, and then to a heterogenous dataset from ambulatory sleep clinic patients. The specific objectives for the evaluation were: (1) Estimate classification performance for sleep-wakefulness detection by accuracy, sensitivity, specificity, and Cohen's kappa statistics using both real-time and future-dependent models, and (2) estimate agreement between methods for the main sleep outcome variable total sleep time (TST) using Bland–Altman analysis, and then similarly for four secondary sleep variables; sleep onset latency (SOL), wake after sleep onset (WASO), sleep efficiency (SE%), and number of awakenings (NW).

## Methods

### Data collection

Two sets of data were collected for this study from different populations. The study protocol for the randomized cross-over trial from which Dataset 1 (DS1) was acquired, and the protocol was approved by the Regional Ethical Committee in Trondheim, (Central Norway; REK: 2017/916) and is registered on the ISRCTN website (reference number 12419665). The study was undertaken in accordance with the Revised Declaration of Geneva [17] and written informed consent was obtained from all participants. The protocol for gathering Dataset 2 (DS2) was approved by the Regional Ethical Committee in Trondheim, (Central Norway; REK: 2017/309). Written informed consent was obtained from all participants.

Both datasets consist of simultaneously collected data from PSG, actigraphy, and IR-UWB radar.

#### PSG

PSG recordings were recorded using Somnomedics Somno HD equipment (Somnomedics GmbH, Randersacker, Germany). Six EEG electrodes were placed according to the International (10–20) system [18]; F3, F4, C3, C4, O1, O2, plus a mastoid reference left side (M1) for the electrodes on the right side and a mastoid reference right side for the left side electrodes (M2); two electrooculografic electrodes (EOG) applied 1 cm laterally and, respectively, 2 cm above and below the right and left lateral eye cantus. EOG-reference electrodes were applied to the left (M1) and the right (M2) mastoids. Surface electromyography (EMG) was registered from the submental and bilateral anterior tibial muscles.

#### Actigraphy

Phillips Actiwatch (Actiwatch Spectrum, Philips Respironics Inc., Murrysville) placed on the nondominant wrist were used for both datasets. Automatic scoring algorithms from the manufacturer were not used in this work. Instead, binned movement data were exported from the actigraphs using Actiware (version 5.70.1; Philips Respironics Inc., Murrysville, PA), and then treated in the same way as the simultaneously recorded radar data. The actigraphy and radar data were subjected to identical sleep/wake classification model development, resulting in models of similar form but with different parameters. These models and their parameters are reported in their entirety in Supplementary Table S1.

#### IR-UWB radar

The radar used was the XeThru model X4M200, a commercially available radar sensor developed by Novelda AS. All radar data for this work were stored in baseband I/Q [19] form, to enable different or improved digital signal processing (DSP) at a later date, and then subjected to the pulse-Doppler signal processing provided by the manufacturer. Their Respiration_2 profile was used, to obtain body movement and respiration rate estimates at a rate of 1 Hz. This profile has a detection zone of 0.40–5.00 m, and a respiration detector range of 8–30 respirations-per-minute (RPM). A more detailed description of the radar can be found in Supplementary Material, or in the manufacturer’s datasheet [20].

### Dataset 1

Dataset 1 (DS1) consists of data from twelve healthy participants of 20–30 years (5 male). These data were collected as part of a randomized cross-over trial meant to evaluate the effect of the light conditions in a state-of-the-art acute psychiatric hospital unit at St. Olavs Hospital, Trondheim, Norway [21]. All 40 patient rooms in this building have radar sensors installed in the ceiling. This study was conducted in the last phase of the building construction period before the unit was opened for patient admissions. Prospective participants were eligible for inclusion if their habitual sleep/wake cycle was normal; i.e. weekday bedtime between 22:30 h and 00:00 h and weekday rise time between 06:30 h and 08:00 h, with small intraindividual variations (<2 h) between weekdays and weekends, and no colour blindness. Exclusion criteria were evidence of any current medical or psychological condition, current use of prescription medication(s), family history of severe mental illness, current sleep problems, night shift work in the preceding 2 years, trans-meridian travel in the preceding 2 months, and/or current use of non-prescription drugs or illicit substances. From September 23, 2017, to October 5, 2017, these participants resided for a total of 10 days in the hospital; five consecutive days in each light environment, with randomized order of exposure and one day of washout in between. Between 08:00 h and 17:00 h participants had to leave the unit to follow their normal daily life of work or studies. They spent the remaining time in the hospital, and from 18:00 h to 07:00 h they were confined to their assigned light environment, with a set bedtime at 23:00 h every night. Each participant wore an Actiwatch set to 15-s epochs every day for the duration of the study, also during the daytime hours. Each room had one radar sensor mounted in the ceiling, and one placed on a nightstand next to the bed. These recorded continuously at 17 frames-per-second (FPS) for the duration of the study. Each participant underwent a total of four nights of PSG; two consecutive nights in each light condition.

### Dataset 2

Dataset 2 was collected to observe a broad cross-section of heterogenous troubled sleepers. From 2017 to 2020, patients referred to the Department of Clinical Neurophysiology at St. Olavs Hospital in Trondheim, Norway, for overnight sleep examination for sleep problems of any kind, could be asked to participate. The only inclusion criterion was their willingness to participate and their informed consent, and there were no exclusion criteria. Participants were outfitted with portable PSG equipment and sent home for ambulatory sleep monitoring, as per standard practice at this department. (Some participants were originally referred to as respiratory polygraphy, a simpler examination than PSG. These patients were “upgraded” to full PSG upon consenting to participate in this study.) In addition, they were given a Phillips Actiwatch actigraph set to 30-s epoch length, and a portable radar sensor configured to record baseband data at 300 FPS—this higher framerate was chosen to enable a more detailed analysis of these recordings at a later date. For the present work, the radar data was downsampled to 17 FPS and processed in the same way as the radar data from DS1. The participants were instructed to place the radar sensor on their nightstand (or on a provided camera stand, if they did not have a nightstand), and to be alone in their bed on the night of the recording.

### Data preparation

Each PSG recording was manually scored by a specialist in clinical neurophysiology according to the AASM Manual for the Scoring of Sleep and Associated Events, version 2.4 [22], and then exported to ASCII-format from the Domino software. The raw radar data was processed using software provided by Novelda AS to output activity and respiration estimates at 1 Hz. Differences in internal clocks for radar, actigraphy, and PSG were corrected with an alignment method based on maximum correlation. To match the length of the PSG epochs, 15-s actigraphy activity counts from DS1 and 1 Hz radar data were aggregated into 30-s bins by taking the mean. The three data types output by the radar digital signal processing [fast movement, slow movement, and respirations per minute (RPM)] were scaled to unify their order of magnitude. Further details about data types and preparations can be found in Supplementary Material.

### Sleep/wake classification model development

The inspiration for the sleep/wake classification approach taken in this work comes from actigraphy. Of the four most common methods for processing wrist actigraphic data from adults, three are based on linear-sliding sum models over a time-horizon [7]. Activity data is binned into epochs of some specific length, commonly 30 s, resulting in a single activity value per epoch. Each epoch is then scored as sleep or wake by comparing a weighted sum of activity values from some “time horizon,” i.e. a number of past, present, and future epochs, to some threshold. These three common methods all use the same time horizon; they score each epoch in the time series by considering its activity value in conjunction with those of the four preceding and two succeeding epochs. Only the parameters of the models are different, optimized for their specific datasets and hardware. One of the three (Rescored Cole–Kripke) also impose an additional layer of post hoc heuristic rules on the output of the initial classifications, which for this work we will call the Cole–Kripke rescoring rules [23, 24].

PSG-scored sleep stages N1, N2, N3, and REM were combined into “sleep” and given the value 0, and epochs with the “wake” state were given the value 1. DS1 was split equally into training and test sets (DS1-train and DS1-test) by assigning the participants to either at random. With the PSG sleep/wake state as the target variable, logistic regression was performed over DS1-train to estimate the parameters of linear sliding-sum models. This procedure was followed for both activity data exported from the actigraphs, and for the radar data. For the radar, each of the three data types (fast movement, slow movement, and RPM) were included separately in the regression, giving three data points per epoch in contrast to the single activity value per epoch used for actigraphy. Every combination of horizon length between zero (only the present) up to and including ten epochs in either direction around the present were considered, for a total of 121 models per sensor. The resulting models output a value between zero and one for each epoch, which can be interpreted as “probability of wake.” A *p* = 0.5 threshold was used on the continuous output values from the regression models to classify each epoch as either sleep or wake. Finally, the Cole–Kripke rescoring rules [23] were applied. For real-time models, the final two rules, (4) and (5), had to be excluded, because they depend on future information. The rules used in our work are: (1) after at least 4 min scored as wake, the next 1 min scored as sleep is rescored wake, (2) after at least 10 min scored as wake, the next 3 min scored as sleep are rescored wake, and (3) after at least 15 min scored as wake, the next 4 min scored as sleep are rescored wake. A more detailed description of the model development can be found in Supplementary Material.

### Classification performance analysis

The classification models were applied to DS1-test, and DS2. Epoch-by-epoch classification performance was evaluated against PSG ground truth by calculating overall accuracy, sensitivity, and specificity values. Because the data contain significantly more epochs of sleep than wake, Cohen's kappa coefficients were also calculated to account for the high probability of correct classification occurring by chance. Cohen's kappa statistic has a range of −1 to 1, where zero indicates agreement equivalent to classification by random chance and κ = 1.00 indicates perfect agreement. Universally accepted guidelines of interpretation for values between zero and one do not exist, so for the purpose of this work we will adopt the categories from Ref. [25]: 0.41 ≤ κ < 0.60 = moderate agreement, 0.61 ≤ κ < 0.80 = substantial agreement, and 0.81 ≤ κ < 0.99 = near-perfect agreement. Forest plots were used to compare the classification performance statistics of the actigraph to those of the radar devices.

### Sleep parameters

Four commonly reported sleep parameters were calculated for all sleep/wake classification results: SOL, the duration between reported bedtime and objectively estimated sleep onset time; TST, the total time spent asleep during a major sleep period; WASO, the total time awake between sleep onset and offset; and SE, the percentage of time spent asleep during a major sleep period. The overall NW during the major sleep period was also counted. For calculating SOL, participants reported their bedtime by pushing a user marker on their PSG equipment. For the nights in DS1 for which no PSG was available, SOL was calculated from the set bedtime at 23:00. For PSG, sleep onset was defined as the first 30-s epoch of any sleep stage after reported bedtime. For both actigraphy and radar, sleep onset was defined as the first epoch of the first three-minute period consecutively scored as sleep, as per the definition used in [26]. Sleep offset, i.e. wake time, was defined as the first epoch after the final epoch scored as sleep. Student's *t*-tests were performed to test the hypothesis that the mean difference between compared modalities and PSG was zero. Cohen's D was used as an effect-size measure, calculated by dividing the difference of the means on the pooled standard deviations. The pooled standard deviations were calculated by averaging the square of the standard deviations and taking the square root of the result [27]. Bland–Altman plots were used for visual comparison of agreements between modalities, plotted with bias and 95% limits of agreement (LA). The regression approach for nonuniform differences was employed to look for proportional bias. When a statistically significant slope was identified, the regression line was included in the Bland–Altman plot, and the *R*^2^ value was reported [28]. Forest plots were used to compare the parameter estimates made with actigraph and radar data, in terms of their absolute difference to corresponding PSG parameters. MATLAB (versions R2018-R2020) was used for all analyses.

## Results

DS1 contains recordings from 12 healthy young adults (mean age ± SD: 23.0 ± 3.1 years, 5 male). An equipment error in one PSG recording and a malfunctioning ceiling radar sensor left 43 nights of triple-registered recordings available for analysis. In total, 126 nights of double-registered nightstand radar and actigraphy data were available, with 117 nights also containing data from the ceiling radar. For actigraphy and the nightstand radar, 24 nights of triple registered data were assigned to DS1-train. For the ceiling radar, two of these nights were missing data, leaving 22 nights. The rest of the data was assigned to DS1-test. DS2 contains triple recorded PSG, nightstand radar, and actigraphy recordings from 28 adult sleep clinic patients (mean age ± SD: 46.25 ± 13.98 years, 19 male). Of these, 13 had obstructive sleep apnea, the rest miscellaneous, and often multiple sleep problems including excessive daytime sleepiness, headaches, restless leg syndrome, and insomnia. A summary of the datasets can be found in Table 1.

**Table 1.** Datasets summary

	Nightstand	Ceiling
Healthy volunteers* – training^†^		
Radar + actigraphy	63	59
Radar + actigraphy + PSG^§^	24	22
Healthy volunteers – test^†^		
Radar + actigraphy	62	58
Radar + actigraphy + PSG^§^	23	21
Patients with sleep disorders^‡^		
Radar + PSG + actigraphy	28	0

Number of concurrent nights of recording performed with three types of sensors/equipment.

*n = 12, mean age ± SD: 23.0 ± 3.1 years, 5 males.

^†^The participants were randomly assigned to a training set for model development, and a testing set for validation.

^‡^Ambulatory sleep disorder patients, mean age ± SD: 46.25 ± 13.98 years, 19 males.

^§^PSG = polysomnography.

Table 2 shows the epoch-by-epoch classification performance of two models, one real-time and one with the four-past-two-future horizon seen in the most common actigraphy algorithms, both with the heuristic Cole–Kripke rescoring rules applied. When multiple nights were collected from each participant in a dataset, values were calculated for all epochs from each participant first, then averaged together. Confusion matrices showing the total number of correctly and incorrectly classified epochs for each model are presented in Table 3. Classification performance of other time horizons with and without rescoring can be found in Supplementary Material. In general, we observed that different time horizons had little effect on model performance beyond a slight decrease as the overall horizon length approached zero. The rescoring rules generally improved the accuracy, specificity, and Cohen's kappa values, at the cost of a slight decrease in sensitivity.

**Table 2.** Classification performance statistics

Model type*	Dataset	Sensor type/placement	Accuracy [%]	Specificity [%]	Sensitivity [%]	Cohen's kappa*100
Four past, two future	Healthy volunteers test set^†^	Radar nightstand	94.8 (1.7)	89.8 (7.1)	96.6 (1.6)	86.9 (5.4)
		Radar ceiling	93.8 (2.3)	86.3 (7.4)	96.6 (2.1)	84.3 (6.6)
		Actigraphy	93.1 (1.2)	85.4 (7.4)	96.0 (2.8)	82.6 (3.2)
Five past, zero future (real time)	Healthy volunteers test set	Radar nightstand	94.5 (1.6)	89.5 (6.4)	96.3 (1.5)	86.3 (4.9)
		Radar ceiling	93.3 (2.4)	85.4 (7.7)	96.3 (1.9)	83.1 (6.8)
		Actigraphy	92.7 (1.1)	83.9 (7.7)	96.1 (2.6)	81.5 (3.2)
Four past, two future	Patients with sleep disorders ^‡^	Radar nightstand	80.9 (15.7)	53.7 (18.4)	89.5 (16.9)	44.8 (25.6)
		Actigraph	83.8 (9.0)	74.3 (20.0)	89.4 (7.3)	53.3 (15.8)
Five past, zero future (real time)	Patients with sleep disorders	Radar nightstand	80.9 (15.3)	53.4 (18.7)	89.7 (16.5)	44.3 (24.8)
		Actigraphy	84.1 (9.0)	74.0 (20.2)	89.9 (6.9)	53.8 (16.2)

Epoch-by-epoch classification performance statistics for two models, both with the heuristic Cole–Kripke rescoring rules applied, compared to PSG^§^-determined sleep/wake. Mean (SD) over the participants in the datasets.

*The models are defined by the number of preceding (past) and succeeding (future) epochs used to score a present epoch.

^†^ *n* = 12, mean age ± SD: 23.0 ± 3.1 years, 5 male, 4 nights of PSG + actigraphy + two radars per participant. The participants were randomly assigned into a training set for model development (*n* = 24/22 for nightstand/ceiling), and a testing set for validation (*n* = 23/21 for nightstand/ceiling). Values were calculated for all epochs from each participant first, then averaged together.

^‡^Ambulatory sleep disorder patients. *n* = 28, mean age ± SD: 46.25 ± 13.98 years, 19 male.

^§^PSG = polysomnography.

**Table 3.** Confusion matrices With four past and two future epochs included in the model

		Radar nightstand		Radar ceiling		Actigraphy	
Healthy volunteers test set*		Wake	Sleep	Wake	Sleep	Wake	Sleep
PSG^†^	Wake	7489	803	6551	1027	6961	1098
	Sleep	708	19 644	667	17 877	845	19 505
TPR/FNR^‡^		0.97/0.03		0.96/0.04		0.96/0.04	
FPR/TNR^§^		0.10/0.90		0.14/0.86		0.14/0.86	
		Radar nightstand				Actigraphy	
Patients with sleep disorders^||^		Wake	Sleep			Wake	Sleep
PSG	Wake	3515	3070			4381	2204
	Sleep	2354	19 123			2352	19 125
TPR/FNR		0.89/0.11				0.89/0.11	
FPR/TNR		0.47/0.53				0.33/0.67	
With five past and zero future epochs included in the model							
		Radar nightstand		Radar ceiling		Actigraphy	
Healthy volunteers test set		Wake	Sleep	Wake	Sleep	Wake	Sleep
PSG	Wake	7465	827	6479	1099	6841	1220
	Sleep	756	19 596	717	17 827	829	19 521
TPR/FNR		0.96/0.04		0.96/0.04		0.96/0.04	
FPR/TNR		0.10/0.90		0.15/0.85		0.15/0.85	
		Radar nightstand				Actigraphy	
		Wake	Sleep			Wake	Sleep
PSG	Wake	3483	3102			4350	2235
	Sleep	2309	19 168			2253	19 224
TPR/FNR		0.89/0.11				0.90/0.10	
FPR/TNR		0.47/0.53				0.34/0.66	

Confusion matrices of epoch-by-epoch classification performance for two selected models, both with the heuristic Cole–Kripke rescoring rules applied, over two datasets. Numbers on the main diagonals of the 2 × 2 matrices represent correctly classified epochs.

*n = 12, mean age ± SD: 23.0 ± 3.1 years, 5 male, 4 nights of PSG + actigraphy + two radars per participant. The participants were randomly assigned into a training set for model development (*n* = 24/22 for nightstand/ceiling), and a testing set for validation (*n* = 23/21 for nightstand/ceiling).

^†^PSG, Polysomnography.

^‡^TPR, true positive rate. FNR, false negative rate.

^§^FPR, false positive rate. TNR, true negative rate.

^||^Ambulatory sleep disorder patients. *n* = 28, mean age ± SD: 46.25 ± 13.98 years, 19 males.

For both datasets, the differences between the real-time model and the non-real-time model were in the sub-percent range for all performance statistics. Across all metrics, the models performed better on DS1-test than on DS2. For the DS1-test, the nightstand radar achieved the best results, followed by the ceiling radar, and then by actigraphy, which outperformed the ceiling radar in terms of specificity but otherwise achieved slightly lower scores. All accuracies were above 92%, all sensitivities above 95%, all specificities above 83%, and all Cohen's kappa values were above 0.81. For DS2 the accuracy of actigraphy was around 83% compared with around 81% for the radar. Both achieved a sensitivity for sleep of approximately 89%, but in specificity for wake and the Cohen's kappa statistic, actigraphy notably outperformed the nightstand radar, with respectively 74% and 0.53 compared with 53% and 0.44.

**Figure 1.**
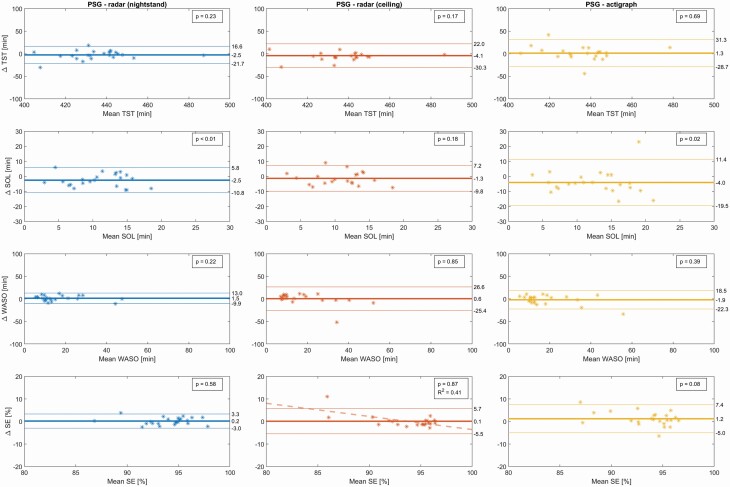
Bland–Altman plots of sleep variables of the test set of healthy volunteers, for the real-time models that score the present epoch by considering it and five past epochs. *p*-values from Student's *t*-test on the hypothesis of the bias being zero. A single significant trend (*p*_slope_ < 0.01) was detected and included with its corresponding *R* squared value. This trend is driven by a single outlying data point. *n* = 12, mean age ± SD: 23.0 ± 3.1 years, 5 male, 4 nights of PSG + actigraphy + two radars per participant. The participants were randomly assigned into a training set for model development (*n* = 24/22 for nightstand/ceiling), and a testing set for validation (*n* = 23/21 for nightstand/ceiling). PSG, polysomnography; SOL, sleep onset latency; TST, total sleep time; WASO, wake after sleep onset; SE, sleep efficiency.

**Figure 2.**
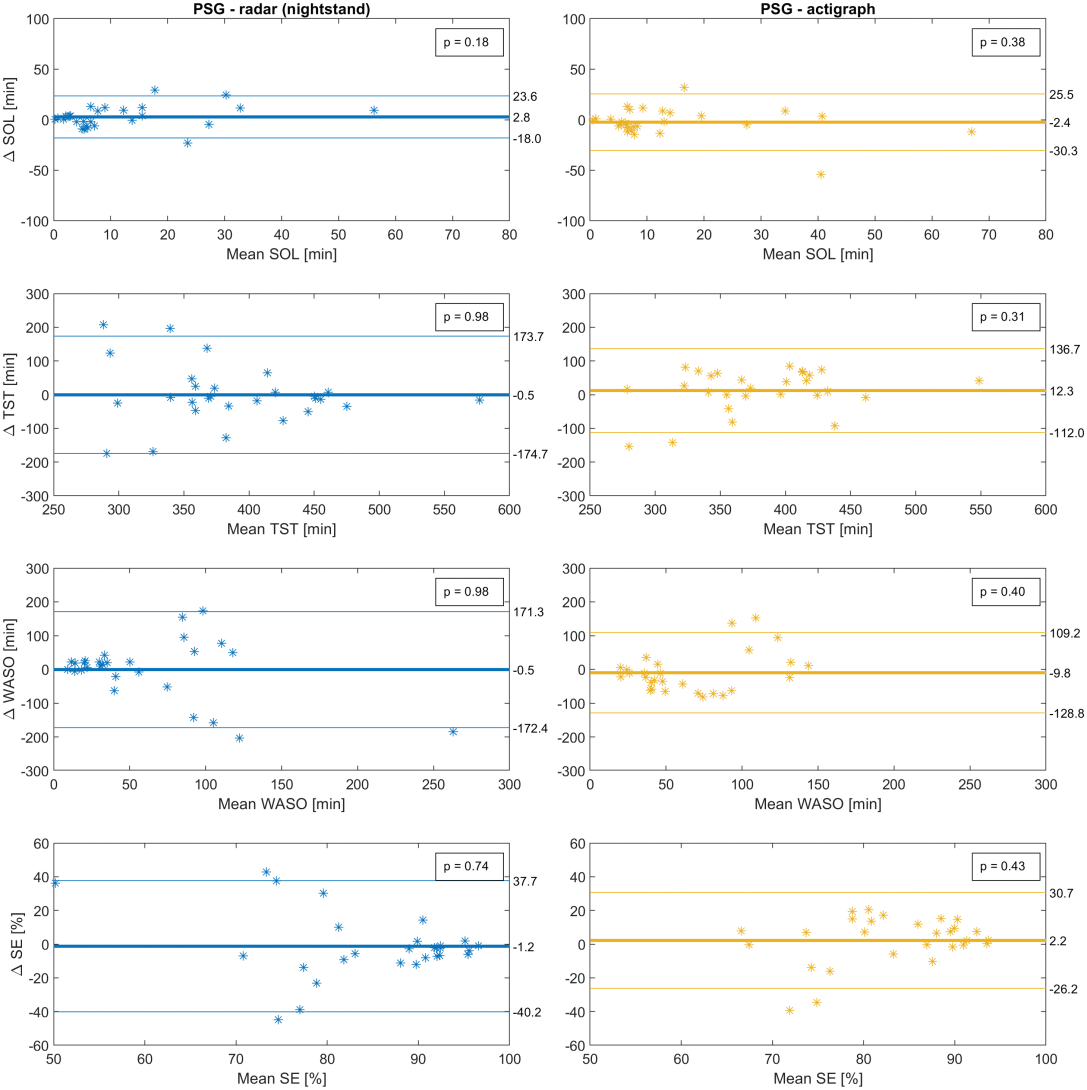
Bland–Altman plots of sleep variables of patients with sleep disorders, for the real-time models that score the present epoch by considering it and five past epochs. *p*-values from Student's *t*-test on the hypothesis of the bias being zero. *n* = 28, mean age ± SD: 46.25 ± 13.98 years, 19 male. PSG, polysomnography; SOL, sleep onset latency; TST, total sleep time; WASO, Wake After Sleep Onset; SE, sleep efficiency.

Sleep variables for the triple-recorded nights for PSG and derived for each sensor for two selected models are shown in Table 4, along with Cohen's D effect sizes and superscripts to indicate parameters significantly different from their PSG counterparts. An expanded version of this table also including the *p*-values can be found in Supplementary Table S6. Bland–Altman plots for the real-time models can be seen in Figures 1 and 2, with their biases and LAs reported in Table 5 along with *t*-tests on the hypothesis of zero bias.

**Table 4.** Sleep parameters

Healthy volunteers test set*								
			Nightstand radar (*n* = 23)		Ceiling radar (*n* = 21)		Actigraphy (*n* = 23)	
Model type^†^	Variable^‡^	PSG^§^ (*n* = 23)	Mean (SD)	Cohen's D	Mean (SD)	Cohen's D	Mean (SD)	Cohen's D
Four past, two future	TST [min]	434.4 (18.2)	437.4 (17.2)	0.17	438.3 (22.6)	0.24	433.2 (20.1)	−0.06
	SOL [min]	10.5 (6.2)	12.6 (6.4)^¶^	0.33	11.0 (6.0)	−0.01	13.7 (6.6)^¶^	0.5
	WASO [min]	17.3 (11.2)	15.1 (13.1)	−0.18	17.5 (17.8)	−0.05	19.3 (17.3)	0.13
	SE [%]	94.0 (2.6)	94.1 (2.9)	0.01	93.9 (4.3)	0.06	93.0 (4.0)	−0.31
	NW [num]	17.6 (4.7)	9.8 (3.9)^#^	−1.8	8.0 (4.7) ^#^	−2.12	12.8 (5.5)^¶^	−0.93
Five past, zero future (real time)	TST [min]	434.4 (18.2)	436.9 (16.4)	0.15	437.3 (21.7)	0.2	433.1 (19.3)	−0.07
	SOL [min]	10.5 (6.2)	13.0 (6.0)^¶^	0.41	12.4 (6.4)	0.21	14.5 (6.5)^¶^	0.63
	WASO [min]	17.3 (11.2)	15.8 (12.1)	−0.13	17.7 (17.0)	−0.04	19.2 (15.8)	0.14
	SE [%]	94.0 (2.6)	93.9 (2.6)	−0.07	93.6 (4.2)	−0.03	92.8 (3.8)	−0.37
	NW [num]	17.6 (4.7)	12.3 (4.9)^#^	−1.09	10.0 (5.3)^#^	−1.57	15.2 (5.7)	−0.46
Patients with sleep disorders^||^								
		PSG (*n* = 28)	Nightstand radar (*n* = 28)				Actigraph (*n* = 28)	
Model type	Variable		Mean (SD)	Cohen's D			Mean (SD)	Cohen's D
Four past, two future	TST [min]	386.7 (73.3)	386.5 (85.2)	0			372.0 (59.3)	−0.22
	SOL [min]	13.0 (14.9)	10.0 (12.4)	−0.22			14.2 (16.1)	0.07
	WASO [min]	61.3 (55.7)	64.9 (77.8)	0.05			74.0 (42.6)	0.26
	SE [%]	84.2 (12.2)	84.7 (16.1)	0.03			81.6 (9.5)	−0.24
	NW [num]	24.6 (13.2)	19.3 (14.0)	−0.39			21.6 (10.5)	−0.25
Five past, zero future (realtime)	TST [min]	386.7 (73.3)	387.2 (85.3)	0.01			374.4 (58.2)	−0.19
	SOL [min]	13.0 (14.9)	10.3 (12.2)	−0.2			15.4 (18.1)	0.14
	WASO [min]	61.3 (55.7)	61.9 (80.8)	0.01			71.2 (39.2)	0.2
	SE [%]	84.2 (12.2)	85.5 (16.2)	0.09			82.0 (9.0)	−0.2
	NW [num]	24.6 (13.2)	23.1 (15.5)	−0.1			23.9 (11.5)	−0.05

Sleep parameters extracted from manually scored hypnograms (PSG) and from the sleep/wake state sequences resulting from the application of two selected classification models to radar and actigraphy data. Sleep onset latency was calculated from a user-marker input. The significant difference found from paired-sample Student's *t*-tests of each model/sensor compared to their corresponding PSG recordings are indicated with superscripts, and the standardized effect sizes are reported by Cohen's D.

*n = 12, mean age ± SD: 23.0 ± 3.1 years, 5 male, 4 nights of PSG + actigraphy + two radars per participant. The participants were randomly assigned into a training set for model development (*n* = 24/22 for nightstand/ceiling), and a testing set for validation (*n* = 23/21 for nightstand/ceiling).

^†^The models are defined by the number of preceding (past) and succeeding (future) epochs used to score a present epoch.

^‡^ SOL, Sleep Onset Latency; TST, Total Sleep Time; WASO, Wake After Sleep Onset; SE, Sleep Efficiency; NW, Number of awakenings.

^§^PSG, Polysomnography (sleep parameters scored manually, independent of models).

^||^Ambulatory sleep disorder patients. *n* = 28, mean age ± SD: 46.25 ± 13.98 years, 19 male.

^¶^ *p* < 0.05 Student's *t*-test, compared to PSG.

^#^ *p* < 0.001.

**Table 5.** Sleep variables from real-time models, compared to PSG*

Healthy volunteers test set^†^		
Variable [units]^‡^	Overall bias (95% CI) [*P*-value]	95% Limits of Agreement
TST [min]		
Radar nightstand	−2.5 (−6.7, 1.7) [0.23]	[−21.7, 16.6]
Radar ceiling	−4.1 (−10.2, 2.0) [0.17]	[−30.3, 22.0]
Actigraphy	1.3 (−5.3, 7.9) [0.69]	[−28.7, 31.3]
SOL [min]		
Radar nightstand	−2.5 (−4.3, −0.7) [0.01]	[−10.8, 5.8]
Radar ceiling	−1.3 (−3.3, 0.7) [0.18]	[−9.8, 7.2]
Actigraphy	−4.0 (−7.5, −0.6) [0.02]	[−19.5, 11.4]
WASO [min]		
Radar nightstand	1.5 (−1.0, 4.1) [0.22]	[−9.9, 13.0]
Radar ceiling	0.6 (−5.5, 6.6) [0.85]	[−25.4, 26.6]
Actigraphy	−1.9 (−6.4, 2.6) [0.39]	[−22.3, 18.5]
SE [%]		
Radar nightstand	0.2 (−0.5, 0.9) [0.58]	[−3.0, 3.3]
Radar ceiling	0.1 (−1.2, 1.4) [0.87]	[−5.5, 5.7]
Actigraphy	1.2 (−0.2, 2.6) [0.08]	[−5.0, 7.4]
NW [num]		
Radar nightstand	5.3 (3.2, 7.3) [<0.001]	[−3.9, 14.5]
Radar ceiling	7.9 (5.7, 10.1) [<0.001]	[−1.4, 17.2]
Actigraph	2.4 (−0.3, 5.1) [0.08]	[−9.9, 14.7]
Patients with sleep disorders^§^		
Variable [units]	Overall bias (95% CI) [*P*-value]	95% Limits of Agreement
TST [min]		
Radar nightstand	−0.5 (−34.9, 34.0) [0.98]	[−174.7, 173.7]
Actigraphy	12.3 (−12.3, 36.9) [0.31]	[−112.0, 136.7]
SOL [min]		
Radar nightstand	2.8 (−1.3, 6.9) [0.18]	[−18.0, 23.6]
Actigraphy	−2.4 (−7.9, 3.1) [0.38]	[−30.3, 25.5]
WASO [min]		
Radar nightstand	−0.5 (−34.5, 33.5) [0.98]	[−172.4, 171.3]
Actigraphy	−9.8 (−33.4, 13.7) [0.40]	[−128.8, 109.2]
SE [%]		
Radar nightstand	−1.2 (−9.0, 6.5) [0.74]	[−40.2, 37.7]
Actigraphy	2.2 (−3.4, 7.8) [0.43]	[−26.3, 30.7]
NW [num]		
Radar nightstand	1.5 (−5.2, 8.2) [0.65]	[−32.2, 35.2]
Actigraphy	0.6 (−4.8, 6.1) [0.81]	[−26.9, 28.1]

Positive values of bias indicate underestimation compared to PSG (*p*-values from Student's *t*-test for the null-hypothesis that the bias is zero). Limits of Agreement given as [bias −1.96*SD, bias +1.96*SD]; SD, standard deviation for paired differences.

*PSG, polysomnography

^†^ *n* = 12, mean age ± SD: 23.0 ± 3.1 years, 5 male, 4 nights of PSG + actigraphy + two radars per participant. The participants were randomly assigned into a training set for model development (*n* = 24/22 for nightstand/ceiling), and a testing set for validation (*n* = 23/21 for nightstand/ceiling).

^‡^TST, Total Sleep Time; SOL, Sleep Onset Latency; WASO, Wake After Sleep Onset; SE, Sleep Efficiency; NW, Number of awakenings

^§^Ambulatory sleep disorder patients. *n* = 28, mean age ± SD: 46.25 ± 13.98 years, 19 male.

For the real-time models applied to DS1-test, all TST estimates were similar to PSG (Table 4) and without significant bias (Table 5). Significant overestimation of SOL by 2.5 and 4.0 minutes (Cohen's D: 0.41 and 0.63) was found for the nightstand radar and actigraphy respectively, with no significant bias found in SOL for the ceiling radar. NW was significantly underestimated by 5.3 and 7.9 discrete awakenings (Cohen's D: −1.09 and −1.57) for the nightstand and the ceiling radars respectively. A single significant slope was detected, for SE compared between PSG and ceiling radar (*R*^2^: 0.41). This slope is driven entirely by a single outlying data point, and disappears when this single point is removed. (The same data point can be observed outside of the LAs in WASO for the same comparates, but does not create a significant trend for this parameter). For the non-real-time models, a significant difference to PSG was found for the same parameters; nightstand SOL (+2.1 min), actigraph SOL (+3.2 min), nightstand NW (−7.8 awakenings), and ceiling NW (−9.7 awakenings). Additionally, the non-real-time model significantly underestimated NW by 4.8 discrete awakenings (Cohen's D: −0.93) for the actigraph. For the same models applied to DS2, TST means were almost identical for PSG and nightstand radar (Table 4), and bias was not present (Table 5). No significant biases were seen in any sleep parameters, but their variations (seen in the standard deviations and confidence interval width) were quite a bit larger than for the DS1-test. In general, we observed good agreement both for TST and the secondary sleep variables.

**Figure 3.**
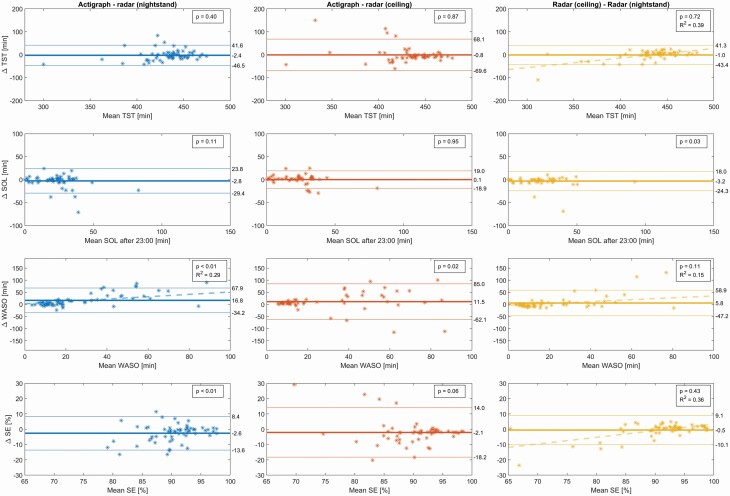
Bland–Altman plots comparing the actigraph and the two radar positions for the test set of healthy volunteers. *p*-values from Student's *t*-test on the hypothesis of the bias being zero. Regression lines with corresponding *R* squared values are included in those subplots wherein a significant (*p*_slope_ < 0.01) trend was detected. *n* = 12, mean age ± SD: 23.0 ± 3.1 years, 5 male, 11 nights of actigraphy + two radars per participant. The participants were randomly assigned to a training set for model development (*n* = 63/59 for nightstand/ceiling), and a testing set for validation (*n* = 63/58 for nightstand/ceiling). SOL, sleep onset latency; TST, total sleep time; WASO, wake after sleep onset; SE, sleep efficiency.

The estimated sleep parameters for all nights of the DS1-test, including those for which PSG was not available, can be seen in Table 6. Bland–Altman plots for the real-time model can be seen in Figure 3, and the biases and LAs are reported in Table 7. No significant differences were found in estimated TST values. A significant difference in SOL of 3.2 minutes was found between the two radar positions, with the nightstand estimating a longer latency than the ceiling positioned sensor. Significant differences were found for both WASO and SE between actigraphy and both radar positions, of 16.8 and 11.5 min WASO and 2.6% and 2.1% SE, respectively, for the nightstand and the ceiling positions. No significant difference was found between the radar positions for these parameters. Significant differences were found between all estimates of NW, of 6.1 and 8.3 discrete awakenings between actigraphy and the nightstand and ceiling radars, respectively, and of 2.8 discrete awakenings between the two radars. Significant slopes indicating the presence of proportional bias were detected for the WASO compared between actigraph and nightstand radar (*R*^2^: 0.29), and for TST, WASO, and SE compared between the ceiling and nightstand radars (*R*^2^: 0.39, 0.15, and 0.36, respectively).

**Table 6.** Sleep parameters estimated with a real-time model over all nights in the test set of healthy volunteers*

	Mean (SD^‡^)		
Sleep parameter^†^ [units]	Actigraphy (*n* = 63)	Nightstand (*n* = 63)	Ceiling (*n* = 58)
TST [min]	431.8 (31.5)	434.2 (26.4)	432.1 (40.2)
SO after 23:00 h [min]	18.2 (14.9)	21.0 (18.7)	17.7 (17.8)
WASO [min]	37.5 (37.3)	20.6 (24.4)	27.0 (34.6)
SE [%]	88.7 (6.8)	91.3 (5.5)	90.7 (8.5)
NW [num]	20.5 (9.0)	14.4 (7.6)	11.9 (7.1)

Sleep parameters extracted from the sleep/wake state sequences resulting from the application of a real-time classification model that includes information from five past and zero future epochs, for all nights in the DS1-test (including the ones without consecutive PSG^§^ recordings). Since no user marker was available for the nights without PSG, sleep onset latency was calculated from the set bedtime at 23:00 h.

*n = 12, mean age ± SD: 23.0 ± 3.1 years, 5 male, 11 nights of actigraphy + two radars per participant. The participants were randomly assigned into a training set for model development (*n* = 63/59 for nightstand/ceiling), and a testing set for validation (*n* = 63/58 for nightstand/ceiling).

^†^TST, Total Sleep Time; SOL, Sleep Onset Latency; WASO, Wake After Sleep Onset; SE, Sleep Efficiency; NW, Number of awakenings.

^‡^SD, standard deviation.

^§^PSG, Polysomnography.

**Table 7.** Sleep variables from real-time models, compared to each other

Healthy volunteers test set*		
Variable [units]^†^	Overall bias (95% CI) [*P*-value]	95% Limits of Agreement
TST [min]		
Actigraphy to radar nightstand	−2.4 (−8.1, 3.3) [0.40]	[−46.5, 41.6]
Actigraph to radar ceiling	−0.8 (−10.1, 8.6) [0.87]	[−69.6, 68.1]
Radars ceiling to nightstand	−1.0 (−6.8, 4.7) [0.72]	[−43.4, 41.3]
SOL [min]		
Actigraphy to radar nightstand	−2.8 (−6.2, 0.7) [0.11]	[−29.4, 23.8]
Actigraphy to radar ceiling	0.1 (−2.5, 2.7) [0.95]	[−18.9, 19.1]
Radars ceiling to nightstand	−3.2 (−6.0, −0.3) [0.03]	[−24.3, 18.0]
WASO [min]		
Actigraphy to radar nightstand	16.8 (10.2, 23.4) [<0.001]	[−34.2, 67.9]
Actigraphy to radar ceiling	11.5 (1.5, 21.4) [0.03]	[−62.1, 85.0]
Radars ceiling to nightstand	5.8 (−1.3, 13.0) [0.11]	[−47.2, 58.9]
SE [%]		
Actigraphy to radar nightstand	−2.6 (−4.0, −1.2) [<0.001]	[−13.6, 8.4]
Actigraphy to radar ceiling	−2.1 (−4.3, 0.1) [0.06]	[−18.2, 14.0]
Radars ceiling to nightstand	−0.5 (−1.8, 0.8) [0.43]	[−10.1, 9.1]
NW [num]		
Actigraphy to radar nightstand	6.1 (4.1, 8.1) [<0.001]	[−9.3, 21.5]
Actigraphy to radar ceiling	8.3 (6.3, 10.4) [<0.001]	[−6.7, 23.3]
Radars ceiling to nightstand	−2.8 (−4.5, −1.1) [0.01]	[−15.2, 9.6]

Sleep parameters extracted from the sleep/wake state sequences resulting from the application of a real-time classification model that includes information from five past and zero future epochs, for all nights in the DS1-test (including the ones without consecutive PSG^‡^ recordings). Since no user marker was available for the nights without PSG, sleep onset latency was calculated from the set bedtime at 23:00 h.

Positive values of bias indicate underestimation by the second sensor relative to the first (*p*-values from Student's *t*-test for the null-hypothesis that the bias is zero). Limits of Agreement given as [bias −1.96*SD, bias +1.96*SD]; SD, standard deviation for paired differences.

*n = 12, mean age ± SD: 23.0 ± 3.1 years, 5 male, 11 nights of actigraphy + two radars per participant. The participants were randomly assigned into a training set for model development (*n* = 63/59 for nightstand/ceiling), and a testing set for validation (*n* = 63/58 for nightstand/ceiling).

^†^SOL, Sleep Onset Latency; TST, Total Sleep Time; WASO, Wake After Sleep Onset; SE, Sleep Efficiency; NW, Number of awakenings.

^‡^PSG, polysomnography.

**Figure 4.**
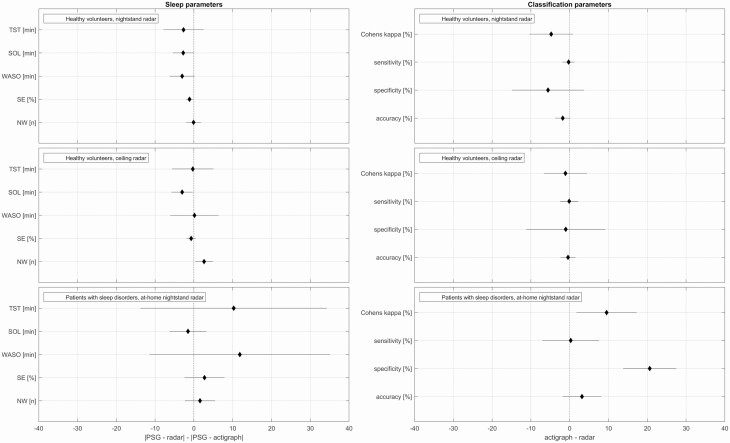
Forest plots comparing the performance of the radar to that of actigraphy, using the real-time models that score the present epoch by considering it and five past epochs. Means and 95% confidence intervals of the differences. Skewing to the left of zero favours the radar. Skewing to the right favours the actigraph. From this figure, it can be inferred that hypothetical non-inferiority margins equal to 20 min for TST and 5 min for SOL would have resulted in conclusions of noninferiority for SOL in all three groups and for TST in healthy volunteers, while noninferiority could not have been claimed for patients since the upper right margin for the CI exceeds 20 min. PSG, polysomnography; SOL, sleep onset latency; TST, total sleep time; WASO, wake after sleep onset; SE, sleep efficiency; NW, number of awakenings.

Forest plots for investigation of the comparative performance of the actigraph versus the radar, using the real-time model, are shown in Figure 4. The first column shows the compared absolute difference between PSG-derived sleep parameters and corresponding parameters estimated from the actigraph and the radar respectively. The second column shows the difference in classification performance statistics between actigraph and radar. Both columns are plotted as means of the differences along with 95% confidence intervals. Skewing left of zero favours the radar and vice versa for the actigraph to the right. For DS1-test in the top two rows, most plotted parameters have means either centred or skewing slightly left, with confidence intervals enveloping zero. The two exceptions both appear for the sleep parameters of the ceiling radar, where SOL falls entirely to the left of the line and NW entirely to the right. For DS2 on the bottom row, the confidence intervals are wider and the means skewing more to the right. All sleep parameters envelop zero in their confidence intervals, but for the classification parameters, the confidence intervals of both Cohen's kappa and specificity fall entirely to the right of the line.

**Figure 5.**
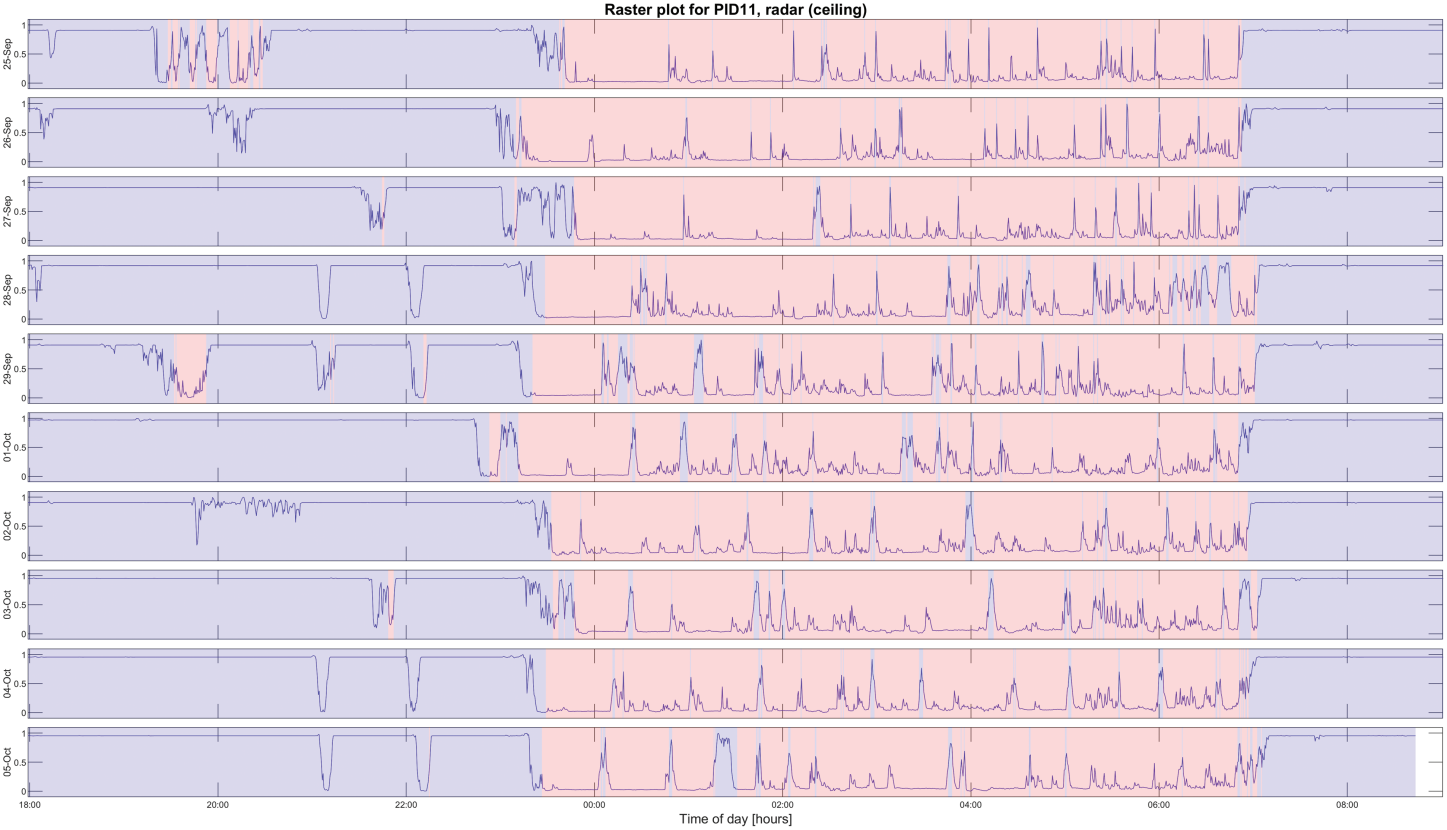
Temporal raster plot generated by the ceiling-mounted radar sensor for a single healthy volunteer. The probability estimate output from the past-five-future-zero regression model is plotted as a line. Each epoch is classified as sleep/wake by comparing the probability estimate to a threshold of *p* = 0.5 and then re-scoring with the Cole–Kripke rules, and the resulting classification is represented by blue (wake) and red (sleep) sections in the raster plot. For reference, this participant underwent PSG on September 28th and 29th, and on October 4th and 5th, achieving a mean (±SD) epoch-by-epoch classification accuracy against PSG sleep/wake of 93.3 (±0.46) percent with corresponding Cohen's kappa values of 0.85 (±0.005) over those days.

Temporal raster plots can be generated to visualize and summarize the patient's stay at the hospital. Figure 5 shows an example for a single participant from DS1, generated from the ceiling-mounted radar. The probability estimate output from the real-time model has been plotted as a line, and the sleep/wake classification result is indicated by the background colour.

## Discussion

Our main results are: (1) Both nightstand and ceiling-mounted radars showed excellent to good agreement with PSG for sleep detection, quite comparable to actigraphy. (2) The performance differences between real-time and non-real-time models were very small. (3) Reliable estimates could be achieved for several standard sleep parameters like TST, SOL, SE%, NW, and WASO. (4) For healthy subjects recorded in a hospital ward environment, an agreement was generally excellent with a small estimated LA. Wider LA was observed for sleep-disorder patients using a nightstand radar in their home bedrooms; comparable with but somewhat larger than LA for actigraphy for the same population. In addition, both actigraphy and radar may tend to overestimate SOL and underestimate nightly awakenings.

For the young healthy sleepers in DS1, the epoch-by-epoch classification results of our models showed high accuracy and sensitivity for both radar sensor placements and the wrist actigraph data, as well as remarkably high specificities and Cohen's kappa values. A lower agreement was observed for the ambulatory sleep-disorder patients in DS2. However, these results are still well within the range of performance commonly reported by previous studies that evaluate actigraphy against PSG over various populations; sensitivities and accuracies for actigraphy tend to lie in the 80%–90% range, with corresponding specificities rarely above 60%, often around 50%, and sometimes even below 30% [7, 10, 26, 29–31].

In general, the real-time models performed slightly worse than the non-real-time models. However, this performance loss is a small cost compared to the benefits of being able to provide an immediate estimate of sleep/wake state. For DS1, the nightstand radar achieved the best overall classification results (larger specificity and kappa values). However, since the differences between radar placements were small, a ceiling-embedded radar might still be preferable to a nightstand mount because it combines greater ease-of-use and flexibility with good performance.

Actigraphy was more specific than nightstand radar in the sleep-disorder patients of DS2. For the sleep parameters, the radars and actigraphy compared with PSG for DS2 showed an excellent similarity of TST, WASO, and SE%. Radars and actigraphy tended to overestimate SOL and to underestimate NW. Comparing the modalities with each other resulted in relative biases on-par with or smaller than those seen in the literature comparing different actigraphy algorithms to each other [7, 31]. For DS2, no statistically significant biases were observed in the estimation of sleep parameters. However, the higher standard deviations and wider 95% confidence intervals, and 95% limits of agreement in the Bland–Altman plots indicate more uncertainty about the estimates for individual subjects recorded at home. The reasons for the large deviations in both directions for TST, WASO, and SE in few ambulatory home recordings should be investigated further, and some possibilities will be discussed in the following paragraphs.

The performances of the radars and the actigraph are compared directly in the forest plots shown in Figure 4. While non-inferiority margins were not prospectively defined for this work (a limitation which is discussed below), this figure shows that most estimated upper 95% confidence interval limits did not exceed hypothetical (but reasonable) non-inferiority margins like 20 minutes for TST and 5 minutes for SOL. Such an analysis would have resulted in conclusions of noninferiority for SOL in all three groups and for TST in healthy volunteers, while noninferiority in TST could not have been claimed for patients since the upper right margin of the confidence interval exceeds 20 minutes. In summary, the large majority of the estimated 95% confidence intervals for sleep and classification parameters showed no difference between radar and actigraphy. There were four exceptions, all for patients with sleep disorders and all skewing in favour of the actigraph: TST, WASO (which is closely related to TST), Cohen's kappa, and specificity.

The discrepancies in results between our two datasets are not unexpected; it is notably more difficult to achieve high classification results and good sleep parameter estimates over a heterogenous set of sleep-disordered patients than over a homogenous set of healthy young volunteers [30]. Subjects who lie quietly but awake in bed for long periods of time pose a challenge to movement-based classifiers, as do subjects with exaggerated movement during sleep. The radar measures movement from the whole body and includes estimated respiration frequency in its classification models. Consequently, unless one can identify and adjust for sleep-disorder specific movements, it is reasonable to expect that conditions like obstructive sleep apnoea (OSA) and restless legs syndrome will cause epoch misclassifications with the current model. It is possible that closer inspection of the probability estimate curves, i.e. the pre-classification model outputs, could be helpful. Whereas a simple *p* = 0.5 classification threshold (with Cole–Kripke rescoring) was sufficient for a population of healthy normal sleepers, a more nuanced approach could be preferable for groups where the level of certainty is lower. Implementing the option of manually correcting classification decisions is another avenue that could be explored, as is the use of adaptive classification thresholds.

A few notes should also be considered when comparing the two datasets considered in this study. Not only is DS1 composed of data from the same demographic as the data on which the classification models were trained; the environment for the training set and the test set was also controlled and identical. These factors are both highly beneficial to classification. In contrast, DS2 was recorded by participants sleeping in their own homes, and they were responsible for mounting the radar sensor on their own. Environmental factors could not be controlled beyond trusting that the participants abided by the requirement of sleeping alone in their beds, and although the digital signal processing done by the radar attempts to compensate for distance to the target, it is possible that inconsistencies in the sensor placement for this dataset had a detrimental influence on the classification performance. This might to some degree explain why the performance difference between the radar and the actigraph was larger for DS2 than for DS1. Further work would be necessary to validate the degree of environmental control needed for optimal results from the radar classifier.

A notable limitation to the present study is that the validation does not include an in-hospital psychiatric population. Sleep in these populations is disrupted and does not necessarily reflect either of the datasets studied in this work. Proceeding with a study of how our method performs in this setting is a natural next step. Furthermore, the datasets examined in the present study do not lend themselves to a thorough examination of possible dependencies of results on factors like age and sex. Future sample selection should be designed following the current recommended guidelines on the development and validation of sleep devices [32].

There are also some limitations to our statistical analyses. Bland–Altman plots were used to illustrate the differences between modalities. However, these have not taken into account that there are uncertainties in the limits of agreement that are difficult to estimate on datasets without more samples [28]. Furthermore, looking at Figures 2 and 3 we observe that there may also be some non-proportional relationships between difference and magnitude; the samples might tend to spread out as TST and %SE decreases, and as WASO increases. A logarithmic transformation prior to plotting could manage this dependency, however since this was not done in comparable studies, it has not been done here [7, 26, 28, 33]. Proportional bias was found only for 5 of 32 B-A plots, and this linearity may seem to be mostly driven by rather few outlying observations. Finally, DS1 lacked a user-marker for bedtime for the non-PSG nights. Calculating sleep onset latency from the set bedtime rather than from a user-marker is not ideal.

Another limitation was the lack of a pre-planned non-inferiority analysis comparing actigraphy and radar [34, 35]. However, there is surprisingly little information and, to our knowledge, no published consensus about the clinically relevant “minimal important difference” (MID) for actigraphic sleep parameters. Studies on actigraphic validity rarely report test–retest (e.g. between-day) differences necessary for the computation of the distribution-based “one standard error of measurement” MID proxy [36–38]. More work is needed to establish a consensus for non-inferiority margins and MIDs for actigraphic sleep parameters.

Furthermore, the data from the radar are rich, and the present work has only considered a small subset of what might be its full potential. In recent years, significant work has been done to develop processing techniques for IR-UWB radar data for non-invasive remote health monitoring [39]. The technology has been used to detect and analyse sleep-disordered breathing [40–42], sleep posture recognition [43], and sleep stage classification [44–47]. O'Hare et.al. [48] compares the sleep assessment performance of two radar-based devices and actigraphy to PSG in a group of twenty healthy subjects. Like us, they observed basically equivalent performance of radar devices and actigraphy for this group. Their reported epoch-by-epoch classification performance was lower than ours (overall accuracies of 85%–86%, Cohen's kappa values of 0.51–0.52), and they observed a statistically significant bias to overestimating sleep time and underestimating WASO and SOL which contrasts our observation of SOL being significantly overestimated. In Pallin et.al. [49], one of these radar devices was found to have similar accuracy to wrist actigraphy for sleep/wake determination in subjects with OSA, with lower sensitivity (86% compared to 94%) and, notably, higher specificity (52% for the radar sensor compared to 34% for actigraphy) and superior estimation of TST, especially at higher apnoea–hypopnea indices. Zaffaroni et al. [50] and Crinion et al. [51] evaluate the same device specifically as a screening tool for OSA, concluding that it is useful particularly for confirming more severe cases. More work is needed to evaluate our radar sensor in the setting of sleep-disordered breathing, but the implication of this in terms of a psychiatric hospital setting is particularly interesting; it indicates a potential for screening of sleep-disordered breathing in a patient population where this traditionally has been challenging.

Interest in non-contact sleep detection and assessment has been growing in popularity over the past decade, and many other methods exist that can be used in ways comparable to the radar. Under-mattress or under-bed sensors have been shown capable of sleep/wake discrimination [52, 53], respiration rate detection [54], and detection of sleep-disordered breathing [55]. The choice of which sensor to use will depend on the circumstances: Nagatomo et al. [56] compares an under-mattress sensor for sleep measurement to PSG in eleven critically ill patients in an intensive care unit (ICU) (achieving agreement, sensitivity, and specificity of 68.4%, 90.1%, and 38.7% respectively). In such a hectic environment, activity in the room might confound a remote-mounted radar sensor, so an under-mattress sensor might be more reliable. On the other hand, an under-mattress system will still require physical sensors and wiring close to the patient, and will only be able to provide measurements while the patient is in their bed. Thus, in a psychiatric hospital setting, a sensor mounted permanently in the ceiling might be preferable.

Infrared camera technology is another tool that has been investigated in this context [57]. The Oxehealth system uses infrared camera technology in combination with computer vision, signal processing, and AI techniques, and has been installed in a psychiatric ward; their work shows that “digitally assisted nursing observation” has potential in terms of improving patient and staff experience at night [58]. However, the use of camera monitoring in such a sensitive environment is potentially problematic. A ceiling-mounted radar sensor could be capable of providing much of the same functionality as a camera-based system, while preserving patient privacy to a greater degree, as it is much more difficult to directly identify individuals from such data.

In summary, radar technology could be used to obtain objective sleep and activity data from certain patient groups from whom it has previously been difficult or even impossible to attain on such a large scale. Since the radar can be embedded in a hospital ceiling or placed on a nightstand for home use, long-term and entirely unobtrusive assessment of sleep in a very wide range of clinical cohorts are possible [59]. In a psychiatric hospital setting, a tool like this could be used to provide night staff with information about the patients' current sleep/wake state, e.g. as displayed on monitors in the staff rooms or sent to hand-held devices, which in turn could improve patient safety at night, reduce the number of nocturnal awakenings due to disturbance from night staff, and lead to more efficient use of limited staff resources. Actigraphy-generated raster plots of the type shown in Figure 5 are known to be very useful in visually depicting changing periodicities associated with circadian dysrhythmia, and they can provide patients with easy to understand graphical displays that may help them to understand diagnostic decisions and evaluate their own response to treatment [60]. Radar-generated plots of this type have the potential to provide many of the same benefits without any potential inconvenience to the patient.

## Conclusions

Our results indicate that our movement-based models can be used with radar data to achieve sleep/wake classification results on par with those normally seen for actigraphy. Our data also supports that the sensor can be used in real-time. Estimates of sleep parameters show little to no bias, although the wide limits of the agreement particularly for the clinical population warn that these still should be interpreted with care. Although our method should be studied further and improved, both to account for various clinical sleep-disorder patient groups and for the heterogeneity in environmental factors of ambulatory home studies, our results show that a non-contact real-time sleep and activity sensor is a real possibility. Accordingly, we believe that a radar-based contact-free sensor has great potential as a supplementary tool in psychiatric ward monitoring and other settings where contact-free sleep monitoring would be advantageous. Such a solution has the potential to change clinical practice in selected fields, improving decision making and care in hospital and home settings, as well as providing a promising new tool for researchers.

## Supplementary Material

zsab060_suppl_Supplementary_Materials

zsab060_suppl_Supplementary_Figure_S1

## Data Availability

Some de-identified data that underlie the results reported in this article may be made available to researchers from accredited research institutions. Data from clinical patients cannot be shared for ethical reasons. Access to data will be limited to investigators who provide a methodologically sound proposal and will be limited to a specified time period (commencing about 3 months after publication of this Article and ending after 5 years). To ensure compliance with the General Data Protection Regulation, data processing must be covered by the European Commission's standard contractual clauses for the transfer of personal data, which must be signed by the data requesters. Proposals and requests for data access should be directed to the corresponding author. *Conflict of interest statement*: As a candidate in the Industrial PhD Scheme the corresponding author (HSAH) is considered an employee both of Novelda AS and the Norwegian University of Science and Technology (NTNU), and receives salary compensation from Novelda AS. None of the other authors have any conflicts of interest to declare.
