# Supplementary material for: Distinguishing sleep from wake with a radar sensor: a contact-free real-time sleep monitor
Source: Sleep. 2021 Jan 8;44(8):zsab060. doi: 10.1093/sleep/zsab060 (PMC8361351; doi:10.1093/sleep/zsab060)
Supplement: zsab060_suppl_Supplementary_Materials [file zsab060_suppl_supplementary_materials.docx]

Distinguishing sleep from wake with a radar sensor

Supplementary materials

Hanne Siri Amdahl Heglum^1,2^

Håvard Kallestad^4, 5^

Daniel Vethe^4, 5^

Knut Langsrud^4, 5^

Trond Sand^1, 3^

Morten Engstrøm^1,3^

^1^ Department of Neuromedicine and Movement Science, Faculty of Medicine and Health Sciences, Norwegian University of Science and Technology (NTNU), Postboks 8905, 7491 Trondheim, Norway

^2^ Novelda AS, Strandveien 43, 7067 Trondheim, Norway

^3^Department of Neurology and Clinical Neurophysiology, St.Olavs University Hospital, Olav Kyrres gate 17, 7030 Trondheim

^4^Department of Mental Health, Norwegian University of Science and Technology, Trondheim, Norway

^5^Division of Mental Health Care, St.Olavs University Hospital, Trondheim, Norway

Corresponding author:

Hanne Siri Amdahl Heglum

Department of Neuromedicine and Movement Science, NTNU

Edvard Griegs gate 8, Trondheim

N-7491 Trondheim

Norway

Phone: +4792852149

Email: [hanne.s.a.heglum@ntnu.no](mailto:hanne.s.a.heglum@ntnu.no)

## Impulse radio ultra-wideband (IR-UWB) radar

An impulse radar generates pulses of radio frequency (RF) signals and measures their reflections from objects in the environment. The term ‘ultra-wideband’ is used to refer to the 3.1-10.6 GHz band of radio frequency waves. The large bandwidth of UWB enables high resolution in spite of very low power, and the high frequencies easily penetrate soft materials such as clothes and beddings while being reflected by more solid objects such as walls and human bodies^1^. With pulse-Doppler signal processing both distance to and velocity of targets can be measured, from the time-of-flight and the Doppler shift of the received signals respectively, and the high resolution means that very fine motions can be detected. The result of this is that the movement of a target within range can be detected without any sensors placed on the target itself.

### XeThru X4M200

The XeThru model X4M200 used in this work is a system on chip (SoC) CMOS IR-UWB radar with built-in TX and RX antennas, developed by Novelda AS. All radar data for this work was stored in ‘raw’ baseband IQ form to enable different or improved digital signal processing (DSP) at a later date. In this work we have employed the pulse-Doppler DSP provided by the manufacturer, specifically their Respiration_2 profile. This profile has a detection zone of 0.40-5.00m, and a respiration detector with a range of detectable respiration-per-minute (RPM) of 8-30RPM^2^.

Static objects in the environment are eliminated by computing a noise map of static reflectors and subtracting these during further processing. The DSP then subjects the data to two parallel sliding-window short-time Fourier transforms; one ‘short’, and one ‘long’. The long window integrates 20 seconds of data. Stable periodic motions that are repeated several times within this time window become amplified – even small and relatively slow oscillations like respiration can be seen clearly in the frequency spectrum. This is then given to a respiration detection algorithm designed to recognize the characteristically symmetrical peaks formed by these movements. Non-repeated fast movements (like a single motion of the arm of a person who is otherwise still) are dampened by the long window, as they get ‘averaged out’. To see these properly, the short window integrates 6 seconds of data and is able to quickly react to fast movements^3^. An index of overall slow and fast body movement is obtained by summarizing the energy of all observed movements over a range covering the whole person (or the closest target, if more than one non-static reflector is observed within the detection zone). Finally, since reflections from further away will appear weaker than reflections from closer targets, the fast and slow movement outputs are normalized by their distance from the radar. The final result is three data types output at a rate of 1Hz: normalized fast movement, normalized slow movement, and RPM.

The XeThru model X4M200 is designed to meet UWB RF specifications of ETSI (Europe), FCC (USA) and ISED (Canada).

## Time alignment

As all three recording types had been running on separate clocks, data had to be time aligned before further analysis could take place. For nights with radar and actigraphy, the radar was aligned to the actigraph. For nights with radar, actigraphy, and PSG, the radar and the actigraph were both aligned to the leg EMG sensors of the PSG. An automated matlab procedure using the xcorr function was used to calculate the time shift of maximum correlation between sensors, and data was shifted by this amount. Every alignment was visually inspected - in most cases the automated procedure identified the visually obvious best result, but in some cases manual adjustment was required due to sensor noise in one of the two PLM-sensors, or due to the procedure locking on the wrong local maximum of the correlation sequence.

The resultant average temporal shifts are indicated in Table S1. An example of movement data before and after alignment, along with the cross-correlation sequences, can be found in supplementary Figure S1.

## Model development

### General formulation

The sleep/wake classification models used in this work were heavily inspired by actigraphy. There are many different actigraphy devices available, using different interpretation schemes, but their basic functionality can usually be described as follows: accelerometer sensor data is recorded, then processed and rate reduced in some way (summation, integration, counting of zero crossings, or some other method) over an epoch of often configurable length (15, 30, or 60 seconds are common). After this, the schemes share the same basic logic: a score of sleep or wake is assigned to an epoch by calculating a weighted sum of activity scores over some time window surrounding that epoch, and then comparing this sum to some threshold. Algorithms with both specific fixed weights^4,5^ and general parametric weights^6^ have been published, where the latter in effect are generalizations of the former.

These models can be summarized as follows:

**Definition 1.** Consider a window of size $\left[ P,F \right]$ surrounding the current time $t$. Let $a[t]$ be a vector of the epoch activity counts within the window $a\left[ t \right]=a\left[ a_{t-P},a_{t-P+1},\ldots a_{t},\ldots,a_{t+F-1},a_{t+F} \right]$. Let $\varphi$ be a (constant) vector of parameters^[[1]](#footnote-2)^, and $S$ some threshold value. Let $0$ represent sleep and $1$ represent wake. A general actigraphy classification function for the epoch at time $t$ can then be written as

$$y\left[ t \right]= \left\{ \begin{aligned} 0, if \varphi a\left[ t \right]=L\left[ t \right]<S \\ 1, otherwise \end{aligned} \right.$$

In the case of the radar data, we expand this general formulation order to include the three data types (slow movement, fast movement, and RPM). A similar expansion could be used i.e. to include data from multiple simultaneous sensors, and/or other features of the data such as measures of variability.

Generate a feature vector $a\left[ t \right], b\left[ t \right], c\left[ t \right],\ldots$ for each property or data type to be included over a time horizon, which does not necessarily have to be of equal length for each feature. Each feature vector has a corresponding vector of parameters $\alpha,\beta,\varphi,\ldots$ , and we expand our model accordingly, including a constant $k$ as an intercept term:

$$L\left[ t \right]= k+ \alpha^{T}a\left[ t \right]+\beta^{T}b\left[ t \right]+\varphi^{T}c\left[ t \right]+\ldots$$

**Definition 2.** Let $x[t]=\left[ 1,{a[t]}^{T},{b[t]}^{T},{c[t]}^{T},\ldots\right]^{T}$be a vector of all the feature vectors we want to include in our model, adopting the convention of letting the first term $x_{0}=1$ to accommodate the intercept. Let the corresponding parameter vector be$\theta=\left[ k,\alpha^{T},\beta^{T},\varphi^{T},\ldots\right]^{T}$.

The sum $L[t]$ can then be written in a condensed form as $L\left[ t \right]=\theta^{T}x[t]$.

### Parameter estimation

In the previous literature on actigraphy the decision about the epoch at time$t$ was made based on how a linear combination $L[t]$ of predictors (features) and parameters $\theta$ compared to some threshold $S$. However, this method does not conserve any information about how close the sum $L[t]$ is to the threshold, nor does it contain any intrinsically obvious interpretation of the meaning of this threshold.

We suggest to let the sum $L\left[ t \right]$represent some version of the probability $p[t]$ of the epoch at time t being scored as wake (the converse $(1-p\left[ t \right])$ represents the probability of the epoch being scored as sleep). Specifically, let us assume that $L[t]$ represents the log-odds of wake:

$$logit\left( p[t] \right)=log\left( \frac{p[t]}{1-p[t]} \right)=\theta^{T}x[t]$$

The probability $p[t]$ is then expressed by the inverse ${logit}^{-1}\left( L[t] \right)$ of the log-odds,

$$p\left[ t \right]=\frac{1}{1+e^{\theta^{T}x[t]}}$$

This function is known as the logistic function, or the sigmoid function. Among many other useful properties, it is the most common hypothesis function used in logistic regression.

The problem of parameter estimation for the models now becomes rather easy. In MATLAB, one can use the fitglm or glmfit functions to solve the problem for a given data set in a single line of code. When the model has been fitted and applied to data, the output will be a continuous value in the range $[0,1]$ that can be interpreted as a probability $p[t]$. To make class predictions, we employed a simple decision rule of a threshold of $0.5$ for each epoch (i.e. choose the class with highest probability for that epoch) in the present study.

### Cole-Kripke rescoring rules

These heuristic rescoring rules act as a nonlinear time series filter on the sleep/wake state sequence, converting initially scored sleep epochs to wake if they occur after or within a specified interval of a given number of awake epochs^7^. First introduced by Webster et al. in 1982^4^, they were concretized and used by Cole et al. ten years later^6^. These rules are:

1. after at least 4 minutes scored as wake, the next 1 minute scored as sleep is rescored wake
2. after at least 10 minutes scored as wake, the next 3 minutes scored as sleep are rescored wake
3. after at least 15 minutes scored as wake, the next 4 minutes scored as sleep are rescored wake
4. 6 minutes or less scored as sleep surrounded by at least 10 minutes of wake (before and after) are rescored wake
5. 10 minutes or less scored as sleep surrounded by at least 20 minutes of wake (before and after) are rescored wake.

For our real-time models, rules d) and e) obviously had to be excluded.

## Additional data preparation

The slow movement and fast movement data types from the radar tend to have values approximately two orders of magnitude larger than the respirations per minute (RPM) estimates from the same. For computational reasons it can be desirable to have the parameter vectors for the data types be of similar magnitudes, so the movement data types were accordingly scaled down by a factor 100 prior to preforming logistic regression, and must of course be correspondingly scaled before applying the resulting models.

## Final models

The feature vectors $x\left[ t \right]$ for the radars were constructed as constructed as

$$x_{radar}\left[ t \right]=\left[ 1,\frac{{fast\_mov[t]}^{T}}{100},\frac{{slow\_mov[t]}^{T}}{100},{RPM[t]}^{T} \right]^{T}$$

For the actigraph, they were simply

$$x_{actigraph}[t]=\left[ 1,{activity[t]}^{T} \right]^{T}$$

The values of the corresponding parameter vectors, $\theta=\left[ k,\alpha^{T},\beta^{T},\varphi^{T} \right]^{T}$ for the radar and $\theta=\left[ k,\alpha^{T} \right]^{T}$ for the actigraph, for the two time horizons presented in the main text ($\left[ -4,2 \right]$ and $\left[ -5,0 \right]$), can be found in table S2.

## Classification performance, expanded tables

This study created models that included 0 to 10 epochs into the past (5 minutes, with 30 second epochs), and 0 to 10 into the future, as well as every possible combination in between, for a total of 121 possible models. The main text considers only the [-4, 2] and [-5, 0] time horizon; supplementary tables S3 and S4 show the classification performance of a wider range of possible time horizons over DS1-train and DS1-test. These results have not been subjected to the Cole-Kripke rescoring rules.

An expanded version of Table 2 from the main text that also includes non-rescored results can be found in supplementary Table S5. An expanded version of Table 4 from the main text that also includes the p-values from Student’s t-tests comparing the estimated sleep parameters to their PSG counterparts can be found in supplementary Table S6.

# References

1. Stone WC. Nist construction automation program report no. 3: Electromagnetic signal attenuation in construction materials. In: Commerce USDo, ed. Gaithersburg, Maryland Building and Fire Research Laboratory, National Institute of Standards and Technology; 1997.

2. Novelda AS. X4M200 Datasheet, Rev. E - Preliminary. 18 Oct 2018; <www.xethru.com>.

3. Wisland DT, Granhaug K, Pleym JR, Andersen N, Støa S, Hjortland HA. Remote Monitoring of Vital Signs Using a CMOS UWB Radar Transceiver. 2016 14th IEEE International New Circuits and Systems Conference (NEWCAS); 26-29 June, 2016; Vancouver, BC, Canada.

4. Webster JB, Kripke DF, Messin S, Mullaney DJ, Wyborney G. An Activity-Based Sleep Monitor System for Ambulatory Use. SLEEP. 1982; 5 (4): 389-399. doi.org/10.1093/sleep/5.4.389.

5. Borazio M, Berlin E, Kücükyildiz N, Scholl P, Laerhoven KV. Towards Benchmarked Sleep Detection with Inertial Wrist-worn Sensing Units. In: proceedings from the 2014 IEEE International Conference on Healthcare Informatics; 2014.

6. Cole RJ, Kripke DF, Gruen W, Mullaney DJ, Gillin JC. Automatic Sleep/Wake Identification From Wrist Activity. SLEEP. 1992; 15 (5): 461-469. doi.org/10.1093/sleep/15.5.461.

7. Haghayegh S, Khoshnevis S, Smolensky MH, Diller KR, Castriotta RJ. Performance comparison of different interpretative algorithms utilized to derive sleep parameters from wrist actigraphy data. Chronobiol Int. 2019; 36 (12): 1752-1760. doi.org/10.1080/07420528.2019.1679826.

# List of Supplementary Figure Captions

## Figure S1

Example of data alignment for a healthy volunteer. For this recording, peaks in the cross-correlation sequences were found at t=-129 seconds for the radar, and t=20 seconds for the actigraph, and the data was correspondingly shifted by these amounts.

# List of Supplementary Table Captions

## Table S1. Average temporal shifts after sensor alignment

Sensors were time-aligned by identifying the distance to maximum cross-correlation of movement and shifting by that amount. Data was aligned to PSG when available. When PSG was not available, radar data was aligned to actigraphy. ^1^ n=12 individuals, mean age ± SD: 23.0 ± 3.1 years, 5 male.

^3^ PSG: Polysomnography

^3^ Ambulatory sleep disorder patients, mean age ± SD: 46.25 ± 13.98 years, 19 male

## Table S2. Parameter vectors for the two models (time horizons [-4, 2] and [-5, 0]) presented in the main text

The probability of wake is calculated according to the formula $p\left[ \boldsymbol{t} \right]\boldsymbol{=}\frac{\boldsymbol{1}}{\boldsymbol{1+}\boldsymbol{e}^{\boldsymbol{\theta}^{\boldsymbol{T}}\boldsymbol{x[t]}}}$, where $\boldsymbol{x[t]}$ is a feature vector of data from the sensor in question.

## Table S3. Classification results over the healthy volunteers training and test sets for a selected range of time horizons

Epoch-by-epoch classification performance statistics for ten selected models. Performance generally increases with the number of epochs included, at the cost of increased complexity in terms of number of parameters.

n=12, mean age ± SD: 23.0 ± 3.1 years, 5 male, 4 nights of PSG + actigraphy + two radars per participant. The participants were randomly assigned into a training set for model development (n=24/22 for nightstand/ceiling), and a testing set for validation (n=23/21 for nightstand/ceiling).

## Table S4. Classification results over the healthy volunteers training and test sets for a selected range of real-time horizons

Epoch-by-epoch classification performance statistics for ten real-time models. Including a longer ‘tail’ of past epochs in the scoring of the present epoch generally increases performance, at the cost of a higher number of parameters in the model.

n=12, mean age ± SD: 23.0 ± 3.1 years, 5 male, 4 nights of PSG + actigraphy + two radars per participant. The participants were randomly assigned into a training set for model development (n=24/22 for nightstand/ceiling), and a testing set for validation (n=23/21 for nightstand/ceiling).

## Table S5. Classification performance, with and without the Cole-Kripke rescoring rules**.**

Epoch-by-epoch classification performance statistics for two models, with and without the heuristic Cole-Krike rescoring rules applied, compared to PSG^3^-determined sleep/wake. Mean (SD) over the participants in the data sets.

The rescoring rules act as a nonlinear time series filter on the sleep/wake state sequence, converting initially scored sleep epochs to wake if they occur after or within a specified interval of a given number of awake epochs. We observe that they generally improve the accuracy, specificity, and Cohen’s kappa values, at the cost of a slight decrease in sensitivity.

^1^ n=12, mean age ± SD: 23.0 ± 3.1 years, 5 male, 4 nights of PSG + actigraphy + two radars per participant. The participants were randomly assigned into a training set for model development (n=24/22 for nightstand/ceiling), and a testing set for validation (n=23/21 for nightstand/ceiling).

^2^ Ambulatory sleep disorder patients. n=28, mean age ± SD: 46.25 ± 13.98 years, 19 male.

^3^ PSG, Polysomnography

| **Table S1.** **Average temporal shifts after sensor alignment** | | | | |
| --- | --- | --- | --- | --- |
| Healthy volunteers^1^  aligned to PSG^2^ | | Nightstand radar | Ceiling radar | Actigraph |
|  | Number of nights (n) | 47 | 43 | 47 |
|  | Avg. temp. shift (seconds) | -139 | -139 | -14 |
| Healthy volunteers  aligned to actigraph | |  |  |  |
|  | Number of nights (n) | 78 | 74 |  |
|  | Avg. temp. shift (seconds) | -188 | -192 |  |
| Patients with sleep disorders^3^  aligned to PSG | |  | | |
|  | Number of nights (n) | 28 |  | 28 |
|  | Avg. temp. shift (seconds) | -150 |  | -134 |
| Sensors were time-aligned by identifying the distance to maximum cross-correlation of movement and shifting by that amount. Data was aligned to PSG when available. When PSG was not available, radar data was aligned to actigraphy. ^1^ n=12 individuals, mean age ± SD: 23.0 ± 3.1 years, 5 male.  ^3^ PSG: Polysomnography  ^3^ Ambulatory sleep disorder patients, mean age ± SD: 46.25 ± 13.98 years, 19 male | | | | |

| Table S2. Parameter vectors for the two models (time horizons [-4, 2] and [-5, 0]) presented in the main text | | | | | | | | |
| --- | --- | --- | --- | --- | --- | --- | --- | --- |
| $\boldsymbol{\theta}\boldsymbol{=}\left[ \boldsymbol{k,}\boldsymbol{\alpha}^{\boldsymbol{T}}\boldsymbol{,}\boldsymbol{\beta}^{\boldsymbol{T}}\boldsymbol{,}\boldsymbol{\varphi}^{\boldsymbol{T}} \right]^{\boldsymbol{T}}$ | | | | | | | | |
| Past 4 future 2 | | | | **Past 5 future 0** | | | | |
|  | **Radar nightstand** | **Radar ceiling** | **Actigraph** |  | **Radar nightstand** | | **Radar ceiling** | **Actigraph** |
| *k* | 2.29541696 | 2.321924235 | -2.537046376 | ***k*** | 2.224988488 | 2.245238055 | | -2.476 |
| $\boldsymbol{\alpha}_{\boldsymbol{T}_{\boldsymbol{0}}\boldsymbol{-4}}$ | 0.02622775 | -0.004505437 | 0.031183202 | $\boldsymbol{\alpha}_{\boldsymbol{T}_{\boldsymbol{0}}-5}$ | 0.018935199 | -0.009281824 | | 0.03211 |
| $\boldsymbol{\alpha}_{\boldsymbol{T}_{\boldsymbol{0}}\boldsymbol{-3}}$ | -0.023148076 | -0.001745622 | 0.0182493 | $\boldsymbol{\alpha}_{\boldsymbol{T}_{\boldsymbol{0}}-\boldsymbol{4}}$ | 0.003475304 | 0.001758058 | | 0.017627 |
| $\boldsymbol{\alpha}_{\boldsymbol{T}_{\boldsymbol{0}}\boldsymbol{-2}}$ | -0.071644213 | -0.001989473 | 0.021077142 | $\boldsymbol{\alpha}_{\boldsymbol{T}_{\boldsymbol{0}}-\boldsymbol{3}}$ | -0.073075754 | -0.007755465 | | 0.01916 |
| $\boldsymbol{\alpha}_{\boldsymbol{T}_{\boldsymbol{0}}\boldsymbol{-1}}$ | -0.150782331 | -0.012469094 | 0.021357249 | $\boldsymbol{\alpha}_{\boldsymbol{T}_{\boldsymbol{0}}-\boldsymbol{2}}$ | -0.083749488 | -0.008823601 | | 0.021216 |
| $\boldsymbol{\alpha}_{\boldsymbol{T}_{\boldsymbol{0}}}$ | 0.21061496 | 0.04178506 | 0.066003221 | $\boldsymbol{\alpha}_{\boldsymbol{T}_{\boldsymbol{0}}-\boldsymbol{1}}$ | -0.169776146 | -0.019129144 | | 0.022211 |
| $\boldsymbol{\alpha}_{\boldsymbol{T}_{\boldsymbol{0}}\boldsymbol{+1}}$ | -0.112249191 | -0.023791666 | 0.013722412 | $\boldsymbol{\alpha}_{\boldsymbol{T}_{\boldsymbol{0}}}$ | 0.156942021 | 0.031064786 | | 0.079858 |
| $\boldsymbol{\alpha}_{\boldsymbol{T}_{\boldsymbol{0}}\boldsymbol{+2}}$ | -0.020656002 | -0.013717754 | 0.026738597 | $\boldsymbol{\beta}_{\boldsymbol{T}_{\boldsymbol{0}}-5}$ | -0.035354117 | -0.019951623 | |  |
| $\boldsymbol{\beta}_{\boldsymbol{T}_{\boldsymbol{0}}\boldsymbol{-4}}$ | 0.003331279 | -0.015442191 |  | $\boldsymbol{\beta}_{\boldsymbol{T}_{\boldsymbol{0}}-\boldsymbol{4}}$ | 0.039690519 | -0.017181948 | |  |
| $\boldsymbol{\beta}_{\boldsymbol{T}_{\boldsymbol{0}}\boldsymbol{-3}}$ | 0.080393217 | -0.006251548 |  | $\boldsymbol{\beta}_{\boldsymbol{T}_{\boldsymbol{0}}-\boldsymbol{3}}$ | 0.074528809 | -0.003136177 | |  |
| $\boldsymbol{\beta}_{\boldsymbol{T}_{\boldsymbol{0}}\boldsymbol{-2}}$ | 0.135411303 | 0.005556379 |  | $\boldsymbol{\beta}_{\boldsymbol{T}_{\boldsymbol{0}}-\boldsymbol{2}}$ | 0.137373216 | 0.011366725 | |  |
| $\boldsymbol{\beta}_{\boldsymbol{T}_{\boldsymbol{0}}\boldsymbol{-1}}$ | 0.239867406 | 0.029647434 |  | $\boldsymbol{\beta}_{\boldsymbol{T}_{\boldsymbol{0}}-\boldsymbol{1}}$ | 0.173963083 | 0.024794082 | |  |
| $\boldsymbol{\beta}_{\boldsymbol{T}_{\boldsymbol{0}}}$ | 0.247616806 | 0.018632057 |  | $\boldsymbol{\beta}_{\boldsymbol{T}_{\boldsymbol{0}}}$ | 0.393472906 | 0.043739137 | |  |
| $\boldsymbol{\beta}_{\boldsymbol{T}_{\boldsymbol{0}}\boldsymbol{+1}}$ | -0.002014974 | -0.007881165 |  | $\varphi_{\boldsymbol{T}_{\boldsymbol{0}}-5}$ | -0.119278492 | -0.102028533 | |  |
| $\boldsymbol{\beta}_{\boldsymbol{T}_{\boldsymbol{0}}\boldsymbol{+2}}$ | 0.0196491 | 0.004586354 |  | $\varphi_{\boldsymbol{T}_{\boldsymbol{0}}-\boldsymbol{4}}$ | -0.049013576 | -0.057099199 | |  |
| $\boldsymbol{\varphi}_{\boldsymbol{T}_{\boldsymbol{0}}\boldsymbol{-4}}$ | -0.106780696 | -0.095298851 |  | $\varphi_{\boldsymbol{T}_{\boldsymbol{0}}-\boldsymbol{3}}$ | -0.057543126 | -0.051653191 | |  |
| $\boldsymbol{\varphi}_{\boldsymbol{T}_{\boldsymbol{0}}\boldsymbol{-3}}$ | -0.043580648 | -0.046111285 |  | $\varphi_{\boldsymbol{T}_{\boldsymbol{0}}-\boldsymbol{2}}$ | -0.05650131 | -0.055757739 | |  |
| $\boldsymbol{\varphi}_{\boldsymbol{T}_{\boldsymbol{0}}\boldsymbol{-2}}$ | -0.048270835 | -0.046182456 |  | $\varphi_{\boldsymbol{T}_{\boldsymbol{0}}-\boldsymbol{1}}$ | -0.061134167 | -0.054659852 | |  |
| $\boldsymbol{\varphi}_{\boldsymbol{T}_{\boldsymbol{0}}\boldsymbol{-1}}$ | -0.051752769 | -0.043281062 |  | $\varphi_{\boldsymbol{T}_{\boldsymbol{0}}}$ | -0.149422212 | -0.139319612 | |  |
| $\boldsymbol{\varphi}_{\boldsymbol{T}_{\boldsymbol{0}}}$ | -0.089895371 | -0.082723051 |  |  |  |  | |  |
| $\boldsymbol{\varphi}_{\boldsymbol{T}_{\boldsymbol{0}}\boldsymbol{+1}}$ | -0.065330391 | -0.072010432 |  |  |  |  |  |  |
| $\boldsymbol{\varphi}_{\boldsymbol{T}_{\boldsymbol{0}}\boldsymbol{+2}}$ | -0.094796015 | -0.082603652 |  |  |  |  |  |  |
| The probability of wake is calculated according to the formula $\boldsymbol{p}\left[ \boldsymbol{t} \right]\boldsymbol{=}\frac{\boldsymbol{1}}{\boldsymbol{1+}\boldsymbol{e}^{\boldsymbol{\theta}^{\boldsymbol{T}}\boldsymbol{x[t]}}}$, where $\boldsymbol{x[t]}$ is a feature vector of data from the sensor in question. | | | | | | | | |

| Table S3. Classification results over the healthy volunteers training and test sets for a selected range of time horizons | | | | | | | | | | |
| --- | --- | --- | --- | --- | --- | --- | --- | --- | --- | --- |
|  | past | future | Error train [%] | Sensitivity training | Specificity training | Cohen’s kappa  training | Error test  [%] | Sensitivity test | Specificity test | Cohen’s kappa test |
| Radar nightstand | 0 | 0 | 9.86 | 0.91 | 0.88 | 0.77 | 8.62 | 0.94 | 0.86 | 0.80 |
|  | 1 | 1 | 7.88 | 0.95 | 0.86 | 0.81 | 6.94 | 0.97 | 0.84 | 0.83 |
|  | 2 | 2 | 7.19 | 0.96 | 0.86 | 0.83 | 6.50 | 0.98 | 0.83 | 0.84 |
|  | 3 | 3 | 6.89 | 0.96 | 0.86 | 0.83 | 6.18 | 0.99 | 0.83 | 0.85 |
|  | 4 | 4 | 6.53 | 0.97 | 0.86 | 0.84 | 6.08 | 0.99 | 0.83 | 0.85 |
|  | 5 | 5 | 6.35 | 0.97 | 0.86 | 0.85 | 5.89 | 0.99 | 0.83 | 0.85 |
|  | 6 | 6 | 6.26 | 0.97 | 0.87 | 0.85 | 5.76 | 0.99 | 0.83 | 0.86 |
|  | 7 | 7 | 6.22 | 0.97 | 0.87 | 0.85 | 5.74 | 0.99 | 0.83 | 0.86 |
|  | 8 | 8 | 6.13 | 0.97 | 0.87 | 0.85 | 5.68 | 0.99 | 0.84 | 0.86 |
|  | 9 | 9 | 6.01 | 0.97 | 0.87 | 0.85 | 5.67 | 0.99 | 0.84 | 0.86 |
|  | 10 | 10 | 5.83 | 0.97 | 0.87 | 0.86 | 5.58 | 0.99 | 0.84 | 0.86 |
| Radar ceiling | 0 | 0 | 10.83 | 0.91 | 0.86 | 0.75 | 9.82 | 0.93 | 0.83 | 0.77 |
|  | 1 | 1 | 8.73 | 0.94 | 0.85 | 0.79 | 8.12 | 0.97 | 0.80 | 0.80 |
|  | 2 | 2 | 8.38 | 0.95 | 0.84 | 0.80 | 7.80 | 0.98 | 0.79 | 0.81 |
|  | 3 | 3 | 8.03 | 0.95 | 0.84 | 0.81 | 7.63 | 0.98 | 0.79 | 0.81 |
|  | 4 | 4 | 7.84 | 0.95 | 0.84 | 0.81 | 7.60 | 0.99 | 0.78 | 0.81 |
|  | 5 | 5 | 7.74 | 0.96 | 0.84 | 0.81 | 7.66 | 0.99 | 0.78 | 0.81 |
|  | 6 | 6 | 7.67 | 0.96 | 0.84 | 0.81 | 7.68 | 0.99 | 0.78 | 0.81 |
|  | 7 | 7 | 7.67 | 0.96 | 0.84 | 0.81 | 7.62 | 0.99 | 0.78 | 0.81 |
|  | 8 | 8 | 7.59 | 0.96 | 0.84 | 0.82 | 7.56 | 0.99 | 0.78 | 0.81 |
|  | 9 | 9 | 7.53 | 0.96 | 0.84 | 0.82 | 7.51 | 0.99 | 0.78 | 0.81 |
|  | 10 | 10 | 7.57 | 0.96 | 0.84 | 0.82 | 7.51 | 0.99 | 0.78 | 0.81 |
| Actigraph | 0 | 0 | 13.24 | 0.98 | 0.61 | 0.65 | 12.67 | 0.13 | 0.98 | 0.62 |
|  | 1 | 1 | 11.76 | 0.97 | 0.67 | 0.70 | 10.91 | 0.11 | 0.98 | 0.69 |
|  | 2 | 2 | 10.69 | 0.97 | 0.70 | 0.73 | 9.65 | 0.10 | 0.98 | 0.73 |
|  | 3 | 3 | 9.81 | 0.97 | 0.73 | 0.75 | 8.85 | 0.09 | 0.98 | 0.75 |
|  | 4 | 4 | 9.10 | 0.98 | 0.75 | 0.77 | 8.19 | 0.08 | 0.98 | 0.77 |
|  | 5 | 5 | 8.55 | 0.98 | 0.77 | 0.78 | 7.76 | 0.08 | 0.98 | 0.79 |
|  | 6 | 6 | 8.18 | 0.98 | 0.78 | 0.79 | 7.37 | 0.07 | 0.98 | 0.80 |
|  | 7 | 7 | 7.72 | 0.98 | 0.79 | 0.81 | 7.02 | 0.07 | 0.98 | 0.81 |
|  | 8 | 8 | 7.34 | 0.98 | 0.80 | 0.82 | 6.72 | 0.07 | 0.98 | 0.82 |
|  | 9 | 9 | 6.95 | 0.98 | 0.82 | 0.83 | 6.41 | 0.06 | 0.98 | 0.83 |
|  | 10 | 10 | 6.65 | 0.98 | 0.82 | 0.84 | 6.26 | 0.06 | 0.98 | 0.84 |
| Epoch-by-epoch classification performance statistics for ten selected models. Performance generally increases with the number of epochs included, at the cost of increased complexity in terms of number of parameters.  n=12, mean age ± SD: 23.0 ± 3.1 years, 5 male, 4 nights of PSG + actigraphy + two radars per participant. The participants were randomly assigned into a training set for model development (n=24/22 for nightstand/ceiling), and a testing set for validation (n=23/21 for nightstand/ceiling). | | | | | | | | | | |

| Table S4. Classification results over the healthy volunteers training and test sets for a selected range of real-time horizons | | | | | | | | | | |
| --- | --- | --- | --- | --- | --- | --- | --- | --- | --- | --- |
|  | past | future | Error train [%] | Sensitivity training | Specificity training | Cohen’s kappa  training | Error test  [%] | Sensitivity test | Specificity test | Cohen’s kappa test |
| Radar nightstand | **0** | **0** | 8.56 | 0.94 | 0.87 | 0.80 | 7.56 | 0.96 | 0.84 | 0.82 |
|  | **1** | **0** | 7.85 | 0.95 | 0.86 | 0.81 | 7.03 | 0.97 | 0.83 | 0.83 |
|  | **2** | **0** | 7.51 | 0.95 | 0.86 | 0.82 | 6.87 | 0.98 | 0.83 | 0.83 |
|  | **3** | **0** | 7.26 | 0.96 | 0.86 | 0.82 | 6.61 | 0.98 | 0.83 | 0.84 |
|  | **4** | **0** | 7.08 | 0.96 | 0.86 | 0.83 | 6.52 | 0.98 | 0.82 | 0.84 |
|  | **5** | **0** | 6.90 | 0.96 | 0.86 | 0.83 | 6.48 | 0.98 | 0.82 | 0.84 |
|  | **6** | **0** | 6.82 | 0.96 | 0.86 | 0.83 | 6.44 | 0.99 | 0.82 | 0.84 |
|  | **7** | **0** | 6.68 | 0.96 | 0.86 | 0.84 | 6.30 | 0.99 | 0.82 | 0.84 |
|  | **8** | **0** | 6.64 | 0.97 | 0.86 | 0.84 | 6.27 | 0.99 | 0.82 | 0.84 |
|  | **9** | **0** | 6.62 | 0.97 | 0.86 | 0.84 | 6.22 | 0.99 | 0.82 | 0.85 |
|  | **10** | **0** | 6.62 | 0.97 | 0.86 | 0.84 | 6.22 | 0.99 | 0.82 | 0.85 |
| Radar ceiling | **0** | **0** | 9.46 | 0.93 | 0.84 | 0.77 | 8.78 | 0.96 | 0.81 | 0.79 |
|  | **1** | **0** | 9.03 | 0.94 | 0.84 | 0.78 | 8.41 | 0.97 | 0.80 | 0.79 |
|  | **2** | **0** | 8.69 | 0.95 | 0.84 | 0.79 | 8.17 | 0.97 | 0.79 | 0.80 |
|  | **3** | **0** | 8.43 | 0.95 | 0.84 | 0.80 | 8.17 | 0.98 | 0.78 | 0.80 |
|  | **4** | **0** | 8.29 | 0.95 | 0.84 | 0.80 | 8.09 | 0.98 | 0.78 | 0.80 |
|  | **5** | **0** | 8.12 | 0.95 | 0.84 | 0.80 | 8.02 | 0.98 | 0.78 | 0.80 |
|  | **6** | **0** | 8.03 | 0.95 | 0.84 | 0.81 | 8.01 | 0.98 | 0.77 | 0.80 |
|  | **7** | **0** | 7.97 | 0.96 | 0.84 | 0.81 | 8.04 | 0.98 | 0.77 | 0.80 |
|  | **8** | **0** | 7.94 | 0.95 | 0.84 | 0.81 | 7.98 | 0.99 | 0.77 | 0.80 |
|  | **9** | **0** | 7.91 | 0.96 | 0.84 | 0.81 | 7.99 | 0.99 | 0.77 | 0.80 |
|  | **10** | **0** | 7.91 | 0.96 | 0.84 | 0.81 | 7.99 | 0.99 | 0.77 | 0.80 |
| Actigraph | **0** | **0** | 12.34 | 13.24 | 0.98 | 0.61 | 0.65 | 12.67 | 0.98 | 0.62 |
|  | **1** | **0** | 11.58 | 12.34 | 0.97 | 0.65 | 0.68 | 11.44 | 0.98 | 0.67 |
|  | **2** | **0** | 10.89 | 11.58 | 0.97 | 0.67 | 0.70 | 10.56 | 0.98 | 0.70 |
|  | **3** | **0** | 10.43 | 10.89 | 0.97 | 0.70 | 0.72 | 9.85 | 0.98 | 0.72 |
|  | **4** | **0** | 9.96 | 10.43 | 0.97 | 0.71 | 0.73 | 9.49 | 0.98 | 0.73 |
|  | **5** | **0** | 9.45 | 9.96 | 0.98 | 0.72 | 0.75 | 8.96 | 0.98 | 0.75 |
|  | **6** | **0** | 9.13 | 9.45 | 0.98 | 0.74 | 0.76 | 8.69 | 0.98 | 0.76 |
|  | **7** | **0** | 8.75 | 9.13 | 0.98 | 0.75 | 0.77 | 8.36 | 0.98 | 0.77 |
|  | **8** | **0** | 8.47 | 8.75 | 0.98 | 0.76 | 0.78 | 8.18 | 0.98 | 0.78 |
|  | **9** | **0** | 8.22 | 8.47 | 0.98 | 0.77 | 0.79 | 7.93 | 0.98 | 0.78 |
|  | **10** | **0** | 8.22 | 8.22 | 0.98 | 0.78 | 0.79 | 7.58 | 0.98 | 0.80 |
| Epoch-by-epoch classification performance statistics for ten real-time models. Including a longer ‘tail’ of past epochs in the scoring of the present epoch generally increases performance, at the cost of a higher number of parameters in the model.  n=12, mean age ± SD: 23.0 ± 3.1 years, 5 male, 4 nights of PSG + actigraphy + two radars per participant. The participants were randomly assigned into a training set for model development (n=24/22 for nightstand/ceiling), and a testing set for validation (n=23/21 for nightstand/ceiling). | | | | | | | | | | |

| **Table S5. Classification performance, with and without the Cole-Kripke rescoring rules.** | | | | | | | |
| --- | --- | --- | --- | --- | --- | --- | --- |
| **Time horizon** | **Rescored** | **Data set** | **Sensor type/placement** | **Accuracy**  **[%]** | **Specificity**  **[%]** | **Sensitivity**  **[%]** | **Cohen’s kappa*100** |
| [-4, 2] | Yes | Healthy volunteers  test set ^1^ | Radar nightstand | 94.7 (2.7) | 88.5 (10.3) | 96.6 (3.2) | 85.8 (8.5) |
|  |  |  | Radar ceiling | 93.6 (3.3) | 84.6 (12.3) | 96.4 (3.8) | 82.5 (10.5) |
|  |  |  | Actigraph | 93.2 (3.0) | 85.1 (11.4) | 95.9 (3.7) | 82.0 (8.0) |
| [-4, 2] | No | Healthy volunteers  test set | Radar nightstand | 92.9 (2.5) | 78.8 (12.2) | 97.9 (2.5) | 80.2 (9.7) |
|  |  |  | Radar ceiling | 91.4 (3.5) | 73.5 (14.5) | 97.7 (3.1) | 75.5 (12.7) |
|  |  |  | Actigraph | 90.6 (2.7) | 71.8 (13.5) | 97.3 (2.6) | 73.5 (9.8) |
| [-5, 0] | Yes | Healthy volunteers  test set | Radar nightstand | 94.5 (2.7) | 88.6 (8.6) | 96.3 (3.1) | 85.6 (7.4) |
|  |  |  | Radar ceiling | 93.1 (3.4) | 83.7 (12.3) | 96.2 (3.7) | 81.3 (10.6) |
|  |  |  | Actigraph | 92.8 (3.0) | 83.8 (10.8) | 96.0 (3.5) | 81.1 (7.4) |
| [-5, 0] | No | Healthy volunteers  test set | Radar nightstand | 92.8 (2.7) | 78.8 (11.5) | 97.7 (2.6) | 80.0 (9.2) |
|  |  |  | Radar ceiling | 91.0 (3.5) | 73.2 (13.8) | 97.4 (3.1) | 74.8 (12.1) |
|  |  |  | Actigraph | 90.5 (2.8) | 71.3 (13.1) | 97.4 (2.5) | 73.2 (9.4) |
| [-4, 2] | Yes | Patients with sleep disorders ^2^ | Radar nightstand | 80.9 (15.7) | 53.7 (18.4) | 89.5 (16.9) | 44.8 (25.6) |
|  |  |  | Actigraph | 83.8 (9.0) | 74.3 (20.0) | 89.4 (7.3) | 53.3 (15.8) |
| [-4, 2] | No | Patients with sleep disorders | Radar nightstand | 81.1 (14.9) | 47.8 (17.0) | 91.4 (14.4) | 42.7 (24.2) |
|  |  |  | Actigraph | 83.6 (9.4) | 65.2 (19.2) | 91.6 (5.6) | 50.3 (14.4) |
| [-5, 0] | Yes | Patients with sleep disorders | Radar nightstand | 80.9 (15.3) | 53.4 (18.7) | 89.7 (16.5) | 44.3 (24.8) |
|  |  |  | Actigraph | 84.1 (9.0) | 74.0 (20.2) | 89.9 (6.9) | 53.8 (16.2) |
| [-5, 0] | No | Patients with sleep disorders | Radar nightstand | 81.0 (14.6) | 48.1 (17.3) | 91.3 (14.1) | 42.2 (23.5) |
|  |  |  | Actigraph | 84.0 (9.4) | 65.6 (19.5) | 92.1 (5.4) | 51.4 (14.9) |
| Epoch-by-epoch classification performance statistics for two models, with and without the heuristic Cole-Krike rescoring rules applied, compared to PSG^3^-determined sleep/wake. Mean (SD) over the participants in the data sets.  The rescoring rules act as a nonlinear time series filter on the sleep/wake state sequence, converting initially scored sleep epochs to wake if they occur after or within a specified interval of a given number of awake epochs. We observe that they generally improve the accuracy, specificity, and Cohen’s kappa values, at the cost of a slight decrease in sensitivity.  ^1^ n=12, mean age ± SD: 23.0 ± 3.1 years, 5 male, 4 nights of PSG + actigraphy + two radars per participant. The participants were randomly assigned into a training set for model development (n=24/22 for nightstand/ceiling), and a testing set for validation (n=23/21 for nightstand/ceiling).  ^2^ Ambulatory sleep disorder patients. n=28, mean age ± SD: 46.25 ± 13.98 years, 19 male.  ^3^ PSG, Polysomnography | | | | | | | |

| **Table S6. Sleep parameters expanded** | | | | | | | | | | | | | | |
| --- | --- | --- | --- | --- | --- | --- | --- | --- | --- | --- | --- | --- | --- | --- |
| Healthy volunteers test set^1^ | | | | | | | | | | | | | | |
| Model^2^ | Variable^3^ | PSG^4^ (n=23) | Nightstand radar (n=23) | | | | | Ceiling radar (n=21) | | | Actigraphy (n=23) | | | |
|  |  | Mean (SD) | Mean (SD) | p | | Cohen’s D | | Mean (SD) | p | Cohen’s D | Mean (SD) | p | | Cohen’s D |
| Four past, two future | TST [min] | 434.4 (18.2) | 437.4 (17.2) | 0.14 | | 0.17 | | 438.3 (22.6) | 0.10 | 0.24 | 433.2 (20.1) | 0.72 | | -0.06 |
|  | SOL [min] | 10.5 (6.2) | 12.6 (6.4) | **0.03** | | 0.33 | | 11.0 (6.0) | 0.93 | -0.01 | 13.7 (6.6) | **0.05** | | 0.5 |
|  | WASO [min] | 17.3 (11.2) | 15.1 (13.1) | 0.10 | | -0.18 | | 17.5 (17.8) | 0.80 | -0.05 | 19.3 (17.3) | 0.41 | | 0.13 |
|  | SE [%] | 94.0 (2.6) | 94.1 (2.9) | 0.92 | | 0.01 | | 93.9 (4.3) | 0.73 | 0.06 | 93.0 (4.0) | 0.14 | | -0.31 |
|  | NW [num] | 17.6 (4.7) | 9.8 (3.9) | **<0.001** | | -1.8 | | 8.0 (4.7) | **<0.001** | -2.12 | 12.8 (5.5) | **0.01** | | -0.93 |
| Five past, zero future (realtime) | TST [min] | 434.4 (18.2) | 436.9 (16.4) | 0.23 | | 0.15 | | 437.3 (21.7) | 0.17 | 0.2 | 433.1 (19.3) | 0.69 | | -0.07 |
|  | SOL [min] | 10.5 (6.2) | 13.0 (6.0) | **0.01** | | 0.41 | | 12.4 (6.4) | 0.18 | 0.21 | 14.5 (6.5) | **0.02** | | 0.63 |
|  | WASO [min] | 17.3 (11.2) | 15.8 (12.1) | 0.22 | | -0.13 | | 17.7 (17.0) | 0.86 | -0.04 | 19.2 (15.8) | 0.39 | | 0.14 |
|  | SE [%] | 94.0 (2.6) | 93.9 (2.6) | 0.58 | | -0.07 | | 93.6 (4.2) | 0.87 | -0.03 | 92.8 (3.8) | 0.08 | | -0.37 |
|  | NW [num] | 17.6 (4.7) | 12.3 (4.9) | **<0.001** | | -1.09 | | 10.0 (5.3) | **<0.001** | -1.57 | 15.2 (5.7) | 0.08 | | -0.46 |
| Patients with sleep disorders^5^ | | | | | | | | | | | | | | |
| Model type | Variable | PSG^2^ (n=28) | Nightstand radar (n=28) | | | | |  | | | Actigraph (n=28) | | | |
|  |  | Mean (SD) | Mean (SD) | | p | | Cohen’s D |  | | | Mean (SD) | p | Cohen’s D | |
| Four past, two future | TST [min] | 386.7 (73.3) | 386.5 (85.2) | | 0.99 | | 0 |  | | | 372.0 (59.3) | 0.24 | -0.22 | |
|  | SOL [min] | 13.0 (14.9) | 10.0 (12.4) | | 0.15 | | -0.22 |  |  |  | 14.2 (16.1) | 0.64 | 0.07 | |
|  | WASO [min] | 61.3 (55.7) | 64.9 (77.8) | | 0.83 | | 0.05 |  |  |  | 74.0 (42.6) | 0.28 | 0.26 | |
|  | SE [%] | 84.2 (12.2) | 84.7 (16.1) | | 0.90 | | 0.03 |  |  |  | 81.6 (9.5) | 0.36 | -0.24 | |
|  | NW [num] | 24.6 (13.2) | 19.3 (14.0) | | 0.08 | | -0.39 |  |  |  | 21.6 (10.5) | 0.26 | -0.25 | |
| Five past, zero future (realtime) | TST [min] | 386.7 (73.3) | 387.2 (85.3) | | 0.98 | | 0.01 |  | | | 374.4 (58.2) | 0.31 | -0.19 | |
|  | SOL [min] | 13.0 (14.9) | 10.3 (12.2) | | 0.18 | | -0.2 |  |  |  | 15.4 (18.1) | 0.38 | 0.14 | |
|  | WASO [min] | 61.3 (55.7) | 61.9 (80.8) | | 0.98 | | 0.01 |  |  |  | 71.2 (39.2) | 0.40 | 0.2 | |
|  | SE [%] | 84.2 (12.2) | 85.5 (16.2) | | 0.74 | | 0.09 |  |  |  | 82.0 (9.0) | 0.43 | -0.2 | |
|  | NW [num] | 24.6 (13.2) | 23.1 (15.5) | | 0.65 | | -0.1 |  |  |  | 23.9 (11.5) | 0.81 | -0.05 | |
| Sleep parameters extracted from manually scored hypnograms (PSG) compared to the sleep/wake state sequences resulting from the application of two selected classification models to radar and actigraphy data. p-values from paired-sample Student’s t-tests of each model/sensor compared to their corresponding PSG recordings show which estimated sleep variables are significantly different from PSG, and the standardized effect sizes are reported by Cohen’s D.  ^1^ n=12, mean age ± SD: 23.0 ± 3.1 years, 5 male, 4 nights of PSG + actigraphy + two radars per participant. The participants were randomly assigned into a training set for model development (n=24/22 for nightstand/ceiling), and a testing set for validation (n=23/21 for nightstand/ceiling).  ^2^ The models are defined by the number of preceding (past) and succeeding (future) epochs used to score a present epoch.  ^3^ SOL, Sleep Onset Latency; TST, Total Sleep Time; WASO, Wake After Sleep Onset; SE, Sleep Efficiency; NW, Number of awakenings  ^4^ PSG, Polysomnography (sleep parameters scored manually, independent of models)  ^5^ Ambulatory sleep disorder patients. n=28, mean age ± SD: 46.25 ± 13.98 years, 19 male. | | | | | | | | | | | | | | |

1. The overall scale factor used by Webster and Cole is simply included into this parameter vector. [↑](#footnote-ref-2)
