# Supplementary figures and images for: Distinguishing sleep from wake with a radar sensor: a contact-free real-time sleep monitor
Source: Sleep. 2021 Jan 8;44(8):zsab060. doi: 10.1093/sleep/zsab060 (PMC8361351; doi:10.1093/sleep/zsab060)

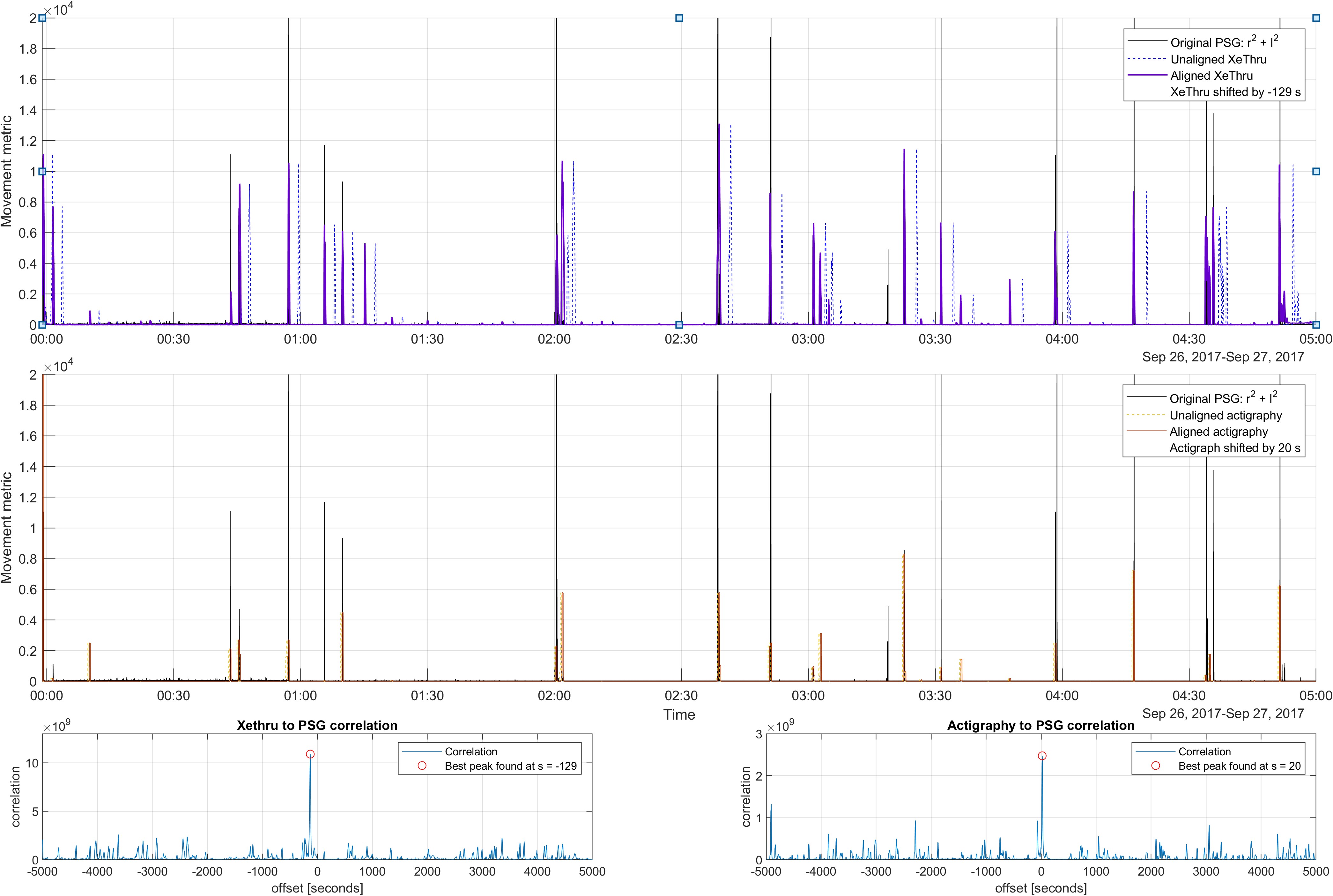

Supplement: zsab060_suppl_Supplementary_Figure_S1 [file zsab060_suppl_supplementary_figure_s1.jpeg]
